# Advances in Colorectal Cancer: Epidemiology, Gender and Sex Differences in Biomarkers and Their Perspectives for Novel Biosensing Detection Methods

**DOI:** 10.3390/ph19010013

**Published:** 2025-12-20

**Authors:** Konstantina K. Georgoulia, Vasileios Tsekouras, Sofia Mavrikou

**Affiliations:** Department of Biotechnology, Faculty of Applied Biology and Biotechnology, Agricultural University of Athens, Iera Odos 75, 11855 Athens, Greece; georgkon.na@aua.gr (K.K.G.);

**Keywords:** colorectal cancer, biosensors, early detection, biomarkers, precision medicine, sex-dependent biomarkers

## Abstract

Colorectal cancer (CRC) remains a major cause of morbidity and mortality worldwide, with its incidence and biological behavior influenced by both genetic and environmental factors. Emerging evidence highlights notable sex differences in CRC, with men generally exhibiting higher incidence rates and poorer prognoses, while women often display stronger immune responses and distinct molecular profiles. Traditional screening tools, such as colonoscopy and fecal-based tests, have improved survival through early detection but are limited by invasiveness, cost, and adherence issues. In this context, biosensors have emerged as innovative diagnostic platforms capable of rapid, sensitive, and non-invasive detection of CRC-associated biomarkers, including genetic, epigenetic, and metabolic alterations. These technologies integrate biological recognition elements with nanomaterials, microfluidics, and digital systems, enabling the analysis of biomarkers such as proteins, nucleic acids, autoantibodies, epigenetic marks, and metabolic or VOC signatures from blood, stool, or breath and supporting point-of-care applications. Electrochemical, optical, piezoelectric, and FET platforms enable label-free or ultrasensitive multiplexed readouts and align with liquid biopsy workflows. Despite challenges related to standardization, robustness in complex matrices, and clinical validation, advances in nanotechnology, multi-analyte biosensing with artificial intelligence are enhancing biosensor performance. Integrating biosensor-based diagnostics with knowledge of sex-specific molecular and hormonal pathways may lead to more precise and equitable approaches in CRC detection, selection of therapeutic regimes and management.

## 1. Introduction

Colorectal cancer (CRC) ranks third in terms of morbidity and fourth in terms of mortality globally, making it one of the most frequently diagnosed cancer types [1]. CRC is a major global health burden, with over 1.9 million new cases and approximately 900,000 deaths annually [2]. In women, it constitutes the second most prevalent malignancy after breast cancer, whereas in men, it represents the third most prevalent after lung and prostate cancer [3,4]. The most common symptoms of CRC are abdominal pain, altered bowel habits, rectal bleeding, and consequent, which can also be found in several gastrointestinal disorders [3]. The location of the alteration determines the clinical manifestations of colorectal cancer [5].

Incidence and mortality are about 25% lower in women than in men. These rates vary geographically as well, with the highest rates found in the most developed countries [6]. Due to the modern lifestyle, colorectal cancer is predicted to increase to 2.5 million new cases worldwide in 2035 in developing countries, whereas stabilizing and declining patterns are observed in highly developed countries. These occurrences have primarily been attributed to national screening programs and a general rise in colonoscopies, but dietary and lifestyle changes may also be pivotal [7]. However, the number of patients under 50 years old who present colorectal cancer, especially left-sided colon cancer and rectal cancer, has increased alarmingly [8,9,10,11]. The specific reasons behind this rise are unclear, although there is a potential correlation with genetic, lifestyle, obesity, and environmental variables. While significant progress has been made in screening programs, early detection remains a challenge due to the asymptomatic nature of early-stage disease. Because of its high incidence and augmented mortality rates, colorectal cancer poses public health issues, raising concerns regarding genetic predisposition, lifestyle and environmental factors. This review aims to provide a comprehensive update on the genetic basis of CRC, molecular subtypes, and emerging biomarkers based on sex differences, for biosensors development with potential clinical applications in early detection, prognosis and treatment selection.

## 2. Epidemiology and Risk Factors

Colorectal cancer (CRC) development is influenced by both genetic and environmental factors. Non-modifiable risk factors include older age, sex, and family history of CRC. Additionally, hereditary syndromes such as Lynch syndrome and familial adenomatous polyposis (FAP) contribute to 5–10% of CRC cases, whereas the remaining 90–95% are classified as sporadic [12,13]. Environmental factors play a crucial role in CRC pathogenesis, as well as obesity, high consumption of red and processed meats, smoking, alcohol intake, and a sedentary lifestyle [14].

Colorectal cancer risk factors can be broadly divided into two categories: non-modifiable and modifiable factors (Table 1) [14,15,16,17,18]. The incidence of colorectal cancer is slightly higher in Black individuals compared to White individuals, although this difference is more likely related to factors such as access to healthcare, screening programs, diet, income, and education, rather than genetics [15]. A recent study by Meester et al. observed an increase in the incidence of early-onset colorectal cancer across multiple racial groups, with a particularly significant rise among non-Hispanic individuals [18].

**Figure 1:**
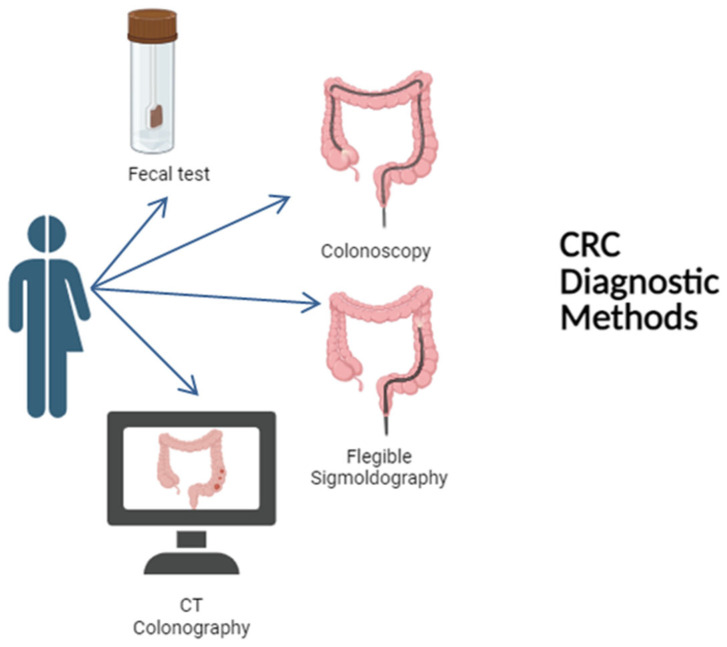
Colorectal cancer (CRC) diagnostic methodologies with a patient-centered approach.

Sex and growing age have continuously demonstrated high correlations with cancer incidence in epidemiological research. The development of CRC is influenced by both environmental and genetic risk factors (Figure 1). Approximately 10–20% of all patients with colorectal cancer appear to have a positive family history [19], with risk varying according to the number and severity of afflicted relatives and the age at which colorectal cancer was diagnosed [20]. The heritability of colorectal cancer is estimated to be between 12% and 35% based on twin and family studies [21,22]. The majority of mechanisms generating heredity are still unknown and need more research, despite the fact that multiple genome-wide association studies of colorectal cancer have effectively identified cancer susceptibility genes (common single-nucleotide polymorphisms) that are linked to colorectal cancer risk [23].

There are two types of hereditary colorectal cancer syndromes: polyposis syndromes and non-polyposis syndromes, which include Lynch syndrome and familial colorectal cancer. Familial CRC affects a subpopulation of 5–7% of people with colorectal cancer [24]. A surveillance plan can be crucial for early diagnosis of CRC patients, providing them with the best therapeutic approach and the right surveillance advice for at—risk family members. Furthermore, patients who have a history of colorectal cancer or adenomas, as well as those who have had inflammatory bowel disease for a long time, are more likely to develop CRC and annual monitoring is recommended [25,26,27].

Polyposis syndromes characterized by numerous lesions are often readily identified. Familial adenomatous polyposis (FAP), for example, can be diagnosed based on polyp count alone. In contrast, Lynch syndrome is characterized by few adenomas that visually resemble sporadic lesions, making clinical recognition difficult. Hence, Lynch syndrome is frequently overlooked without further molecular evaluation [28].

To improve early diagnosis, guidelines currently suggest molecular testing of tissue biopsies in all CRC patients, especially those under 70 years old, as part of systematic screening protocols. Standard approaches include microsatellite instability (MSI) testing and immunohistochemistry (IHC) for mismatch repair (MMR) proteins. These methods serve as initial triage prior to germline genetic testing [29,30]. MSI, a hallmark of MMR deficiency characteristic to Lynch syndrome—associated tumors, is denoted by the expansion or contraction of microsatellite loci in tumor DNA in comparison with normal tissue [31]. Lynch-associated CRC tumors are typically MSI-high, reflecting the underlying germline defects in MMR genes.

Additionally, immunohistochemistry reveals a lack of mismatch repair proteins in these tumors. Nevertheless, MSI is not exclusive to Lynch syndrome, and it is also present in about 15% of sporadic colorectal malignancies. Patients with Lynch syndrome are recommended to undergo frequent colonoscopies, one to two per year, between the ages of 20 and 25 due to the accelerated adenoma–carcinoma pathway [24,32]. Moreover, endometrial cancer and other cancers (such as those of the small intestine, stomach, ovaries, renal pelvis, urethra and hepatobiliary system) are more likely to occur in these patients. The risk of CRC is enhanced by a number of environmental lifestyle variables that are modifiable, including smoking, excessive alcohol use, increasing body weight, and consumption of red and processed meat [33].

## 3. CRC Staging

Pierre Denoix created the tumor, lymph node, metastasis (TNM) nomenclature in the 1940s and 1950s, and the Union for International Cancer Control (UICC) released staging recommendations based on this nomenclature in 1958 [34,35]. The American Joint Committee on Cancer (AJCC) (Table 2) and the UICC have now released joint consensus guides using this standard nomenclature, placing colorectal cancer diagnosis, treatment, and prognosis in the context of other malignancies found throughout the human body. Once tissue is available, pathological staging (pTNM) typically takes precedence over imaging, which is utilized for clinical (cTNM) or post-therapeutic (ycTNM) staging. Based on the maximum degree of local tumor invasion into the adventitia (T3), visceral peritoneum (T4a, if intraperitoneal), muscularis propria (T2), submucosa (Tis or T1), or beyond into other structures (T4b), the T component is determined. The number of positive regional nodes up to ≥7 is reflected in the N stage (N4b). The number of other solid organs involved up to ≥2 (M2) is specified by M staging. As new information becomes available, these criteria are updated frequently. Most recently, stage M1c was added to define peritoneal illness, and stage N1c was given further specifics to identify non-nodal regional tumor deposits [36]. As with other malignancies, colorectal cancer stages are categorized into prognostic stage groups I–IV, with lettered subgroups meant to express increasingly poorer prognosis.

### 3.1. Polyps of the Colon

Colorectal cancer (CRC) typically develops through a well-established adenoma-carcinoma sequence, in which benign polyps undergo a series of genetic and histological changes before transforming into malignant tumors. A polyp of the gastrointestinal tract is defined as a mass that projects into the lumen of the intestine.

In their early stages, polyps often do not present symptoms, which makes them difficult to detect without regular screening. Nonetheless, their existence—particularly when it comes to large quantities or size—represents an essential initial indicator of neoplastic transformation in the colonic epithelium. Research has shown that over 95% of colorectal cancers develop from preexisting adenomas, and the risk of malignancy is closely linked to the size of the polyp, its histologic type (adenomatous, villous or tubular), and the level of dysplasia [37].

### 3.2. Adenomatous Polyps

Adenomatous polyps are benign neoplasms originating from the mucosal epithelium. They consist of crypt cells that have formed clusters on the surface, exceeding the normal number. Many are located in the rectosigmoid region and can be detected through digital rectal examination or sigmoidoscopy, while others may be found throughout the colon [38].

### 3.3. Tubular Adenomas

These polyps constitute approximately two-thirds of benign colonic polyps. They are spherical with smooth borders and typically measure less than 2 cm in diameter. About 4% exceed 2 cm. They exhibit minor epithelial atypia but can present significant dysplastic changes, progressing to invasive cancer in larger polyps. These dysplastic intramucosal lesions are classified as in situ carcinomas. Any dysplastic lesion not infiltrating the muscularis mucosae is considered surgically curable [39].

### 3.4. Villous Adenomas

These polyps are usually located in the rectosigmoid region. They are large masses projecting into the intestinal lumen with a cauliflower-like appearance. Their size exceeds 2 cm, with diameters potentially reaching 10–15 cm. Unlike tubular adenomas, villous adenomas often contain carcinomatous foci. Therefore, colorectal cancer often develops in areas where polyps previously existed [39].

## 4. Biomarkers of CRC

### 4.1. Genetic Markers

Deciphering the human genome has enabled the detection of cancer-related genetic changes in cancer with unprecedented precision. Through a systematic examination of these changes, the sequences of human protein-coding genes with well-defined functions have been determined. Due to the high level of genetic variation in colorectal cancer, determining the clinical significance of specific mutations remains challenging. Evidence suggests that some previously considered rare mutations in colon cancer are actually more common than previously thought and may contribute to the development of other cancers [40,41].

This discovery has opened new avenues for tumor biology research and established novel targets for diagnostics and treatment [42]. Cellular integrity depends on genome stability, and genetic instability leads to various alterations in chromosome structure. The loss of the wild-type allele of tumor suppressor genes such as *APC*, *P53*, and *SMAD4*, which normally inhibit malignant transformation, plays a crucial role in tumor development. Chromosomal instability drives the initiation, promotion, and progression of tumors, involving environmental factors, hereditary predispositions, and acquired somatic mutations in colorectal epithelial cells.

To date, eleven somatic mutations in these genes have been reported in approximately 130 cases of colorectal cancer. Errors in the DNA sequence of colorectal cancer patients lead to the inactivation of repair genes, known as mismatch repair (MMR) genes. These errors can be either inherited (as in hereditary non-polyposis colorectal cancer, HNPCC) or acquired [43]. Lynch syndrome, caused by mutations in *MLH1*, *MSH2*, *MSH6*, and *PMS2*, increases susceptibility to cancer. Each year, more than one million patients are diagnosed with colorectal cancer, and approximately 3% of them have Lynch syndrome, which predisposes them to developing HNPCC.

Oncogenes are genes whose activation contributes to the transformation of normal cells into cancerous cells, whereas tumor suppressor genes encode proteins responsible for maintaining normal cellular function. Oncogenes associated with colorectal cancer include *Ras*, *EGFR* (*Erb-B1*), *Erb-B2*, *TGF-α*, and *TGF-β1.* Tumor suppressor genes involved in colorectal cancer include *APC*, *p53, p27*, *MSI*, *LOH 18q*, the 5q allele deletion, and DNA hypermethylation [44]. Among oncogenes, *Ras* is particularly significant, as its activation promotes cell proliferation and is believed to be an early event in oncogenesis. In colorectal cancer, Ras gene mutations contribute to approximately 40–50% of cases by encoding proteins located on the inner surface of the plasma membrane that bind guanine nucleotides and exhibit GTPase activity. In addition to oncogenes, tumor suppressor genes—most notably *TP53*—play a critical role in solid tumors.

Recent molecular studies have revealed sex-specific pathways that contribute to CRC pathogenesis. A key biomarker is the Y-linked gene KDM5D, which shows significant upregulation in male patients with *KRAS*-mutant tumors. *KDM5D* promotes tumor aggressiveness by weakening cell adhesion and dampening immune responses, thereby explaining the poorer prognosis often seen in men [45]. Conversely, estrogens appear to confer a protective role in females. Estrogen receptor beta (*ERβ*), highly expressed in normal colonic epithelium, is downregulated in CRC tissues, especially in male patients. Its loss is associated with increased inflammation, dysbiosis, and tumor progression. In women, estrogens also influence the gut microbiome to develop a profile that diminishes carcinogenic potential and enhances metabolic health [46].

Further sex-influenced biomarkers consist of inflammatory and immune markers such as IL-6, C-reactive protein (CRP), and tumor-infiltrating lymphocytes (TILs), with females exhibiting stronger immune surveillance [11]. Moreover epigenetic mutations—like the methylation of genes such as *MLH1*, *CDKN2A*, and *APC*—that exhibit sex—dependent patterns and that affect the onset and development of CRC, as they are presented in Table 3 [47].

### 4.2. Inflammatory Markers

Inflammatory markers such as interleukin-6 (IL-6), C-reactive protein (CRP) and tumor immune infiltration (tumor-infiltrating lymphocytes, TILs) counts show sex-dependent patterns in colorectal cancer. Several recent reviews and cohort studies report that female CRC patients tend to display a more activated T cell compartment in the tumor microenvironment (higher activation of CD4^+^ and CD8^+^ subsets and more activated lymphocytes), a profile that has been proposed to reflect stronger immune surveillance in women and which may contribute to sex differences in outcome [46,48,49].

IL-6 is a pleiotropic cytokine that modulates inflammation, tumor cell proliferation and systemic responses; IL-6 signaling and its downstream effects can differ between sexes because of interactions with sex hormones and sex-specific receptor signaling, potentially altering tumor behavior and cachexia syndromes in a sex-dependent way. Genetic and population studies also point to sex interactions for CRP (both genetically determined CRP levels and measured CRP) in relation to CRC prognosis, although results have been heterogeneous across cohorts [50,51]. Elevated IL-6 and a systemic inflammatory profile are often associated with more aggressive disease, but the prognostic role of circulating CRP in CRC is inconsistent between studies and sex can modify these associations, meaning CRP alone is an imperfect biomarker unless analyzed with sex-stratified models or combined immune/tumor markers. Importantly, robust prognostic value is clearer for intratumoural immune markers, particularly in microsatellite-instable (MSI-high) tumors, where high TILs predict better outcomes and may underlie part of the observed female survival advantage [52,53].

### 4.3. Sex-Specific Biomarkers

It is fundamental to note that there is vast evidence that sex affects CRC development. Epidemiological data consistently show that men are diagnosed more frequently and at a younger age than women, while postmenopausal women often present with right-sided or MSI-high tumors. These differences are not only attributed to lifestyle or hormonal factors but also to underlying molecular and epigenetic mechanisms that shape tumor biology differently between sexes. Understanding these differences and incorporating them into risk models could improve both prevention and therapeutic outcomes. Customized strategies may encompass microbiota modulation, hormonal therapy, and improved screening campaigns aimed at men, who are currently underserved in existing preventive programs [54]. Epigenetic modifications, specifically DNA methylation changes, and variations between sexes play a role in cancer research.

It is well-established that promoter hypermethylation occurs in DNA repair (MLH1), cell-cycle regulator (CDKN2A/p16), and tumor suppressor (APC) genes in colorectal cancer (CRC) [55]. Numerous molecular studies and reviews indicate that the frequency of some of these methylation changes is dependent on sex: particularly, hypermethylation of the MLH1 promoter, leading to a sporadic MSI-high phenotype, has been more frequently reported among female patients in various cohorts (which accounts for a higher prevalence of MSI-high tumors in older women). In contrast, reports regarding methylation of CDKN2A and APC show considerable variability across different studies and populations [56]. Some data suggest slightly higher methylation rates of CDKN2A in males, while APC methylation appears relatively balanced between sexes but remains crucial for early adenoma formation [57]. Beyond their oncogenic effects, these methylation events influence immune activity by altering the tumor’s neoantigen landscape and its interaction with tumor-infiltrating lymphocytes (TILs). MSI-high tumors, often arising through MLH1 hypermethylation, display increased immune infiltration and distinct antigenic profiles, which contribute to their better prognosis and higher responsiveness to immunotherapy—phenomena more frequently observed in female patients [58,59,60].

Collectively, these findings emphasize that sex is not merely an epidemiological factor but a biological variable influencing CRC initiation, molecular evolution, and immune environment. Table 3 summarizes a selection of biomarkers for which sex-related associations have been described in CRC. It lists each biomarker’s biological function, how its behavior varies by sex, age at diagnosis and stage association [49]. It highlights both genetic and immunological elements—ranging from Y-linked enzymes like KDM5D to hormonal regulators such as Estrogen Receptor β (ERβ) and inflammatory markers (IL-6/CRP) [61]. By linking these biomarkers to patient sex, age, and tumor stage, the table underscores how biological sex modulates CRC development at multiple levels: genetic, epigenetic, and immune [62]. This overview provides a framework for integrating sex-specific biomarkers into future screening strategies and personalized treatment models.

**Table 3:** Sex-Associated Biomarkers in CRC with Diagnostic Age and Stage.

Biomarker	Biological Role	Sex Association	Age of Diagnosis	CRC Stage Association
KDM5D [45]	Y-linked histone demethylase; regulates chromatin and immune evasion	Male-specific (Y chromosome)	Frequently <60 years (early-onset CRC)	Advanced stages (III–IV); linked to metastasis
Estrogen Receptor β (ERβ) [63]	Tumor suppressor; maintains epithelial homeostasis	Higher in females	Protective in premenopausal women	Downregulated in CRC; more suppressed in males
MLH1 (Methylation) [64]	Mismatch repair gene; silencing leads to microsatellite instability	More common in females >60	Later onset (>60 years)	Frequently stage II–III in right-sided tumors
CDKN2A (p16) Methylation [65]	Tumor suppressor; involved in cell cycle arrest	Slightly higher in males	>50 years	Often in early-stage tumors; associated with serrated pathway
APC Mutations [66]	Wnt pathway regulator; initiates adenoma-carcinoma sequence	Present in both sexes	40–70 years	Early stage (I–II); key in adenoma formation
IL-6/CRP [67]	Inflammatory cytokines; reflect tumor-promoting inflammation	Elevated more in males	Often associated with advanced age	Correlates with advanced stage and poor prognosis
TILs (Tumor-Infiltrating Lymphocytes) [68]	Reflect host immune response	More abundant in females	Variable	Associated with better outcomes in early to intermediate stages

### 4.4. Biomarkers Affected by Lifestyle and Environmental Factors

Lifestyle and environmental factors have a crucial role in tumor initiation as well as progression. A diet rich in red meat, low in fiber or fruits and vegetables, is highly associated with an increase in CRC risk, due to the carcinogenic compounds produced by cooking, such as nitrosamines. In addition, obesity can lead to diabetes, insulin resistance, or chronic inflammation, altering gut microbiota, which are in favor of colorectal carcinogenesis. Furthermore, chronic tobacco use and alcohol consumption exacerbate DNA damage and oxidative stress within epithelial cells of the colon. A well-balanced diet with regular physical activity and stable body mass are recognized as protective factors for CRC. While these general lifestyle determinants are well established, recent studies suggest that gender specific factors may also influence the progression of the disease, giving the spark for further studies [69,70]. Alcohol consumption and smoking pose a relatively higher risk of CRC in men, while obesity and type 2 diabetes demonstrate stronger links in post-menopausal women, possibly due to interactions with insulin resistance and hormonal shifts [71].

Recent epidemiological studies have correlated the contribution of risk factors to variant age groups. In younger adults (20–39 years), behavioral determinants such as tobacco smoking and alcohol consumption were the predominant contributors to colorectal cancer risk. Among middle-aged individuals (40–59 years), stronger associations were observed with obesity-related factors, whereas in older adults (≥60 years), chronic comorbidities—particularly diabetes and hypertension—were the most influential risk determinants. The composite risk scores and their corresponding cut-off thresholds offer evidence-based guidance for implementing age-tailored lifestyle interventions in colorectal cancer prevention strategies [72].

## 5. CRC Diagnostic Methodologies

Colorectal cancer screening has proven to be a cornerstone in the prevention and early detection of this disease (Figure 1). Regular screening, combined with education on the importance of these tests, can significantly reduce mortality rates and enhance quality of life. By identifying precancerous polyps (adenomas) and early-stage cancers, interventions such as polypectomy or early treatment can prevent their progression to more advanced stages of cancer. Early detection not only improves survival rates but also helps reduce treatment costs and the overall burden on healthcare systems.

Colorectal cancer screening is generally recommended to begin at age 45 for individuals at average risk, as the incidence of CRC rises significantly after this age. Those with a family history of colorectal cancer, genetic predispositions such as Lynch syndrome or familial adenomatous polyposis, or personal history of inflammatory bowel disease should begin screening earlier and may require more frequent surveillance.

Symptoms observed in the general population, suggesting the presence of CRC—either benign or malignant—are rectal bleeding, abdominal pain and altered bowel habit. However, the clinical significance of these symptoms is often overlooked.

### 5.1. FOBT and FIT

The Fecal Occult Blood Test (FOBT) and Fecal Immunochemical Test (FIT) are non-invasive stool-based screening tools for colorectal cancer (CRC) that identify hidden blood in feces. The guaiac-based FOBT (gFOBT) detects the heme component of hemoglobin through a chemical reaction with guaiac resin, requiring dietary restrictions to reduce false-positive results. Conversely, FIT utilizes antibodies that specifically bind to human hemoglobin, offering greater sensitivity and specificity for lower gastrointestinal bleeding without dietary limitations. Due to its superior accuracy, ease of use, and the need for only a single stool sample, FIT is generally preferred over gFOBT, which often requires multiple samples for reliable detection [73].

Both tests are recommended as annual screening methods for individuals at average risk of CRC, with positive findings necessitating a follow-up colonoscopy for definitive diagnosis. While effective for early detection, both tests can directly identify polyps and both are subject to false-positive and false-negative results. Nonetheless, their affordability, accessibility, and non-invasive nature make them essential tools in CRC screening programs and prevention strategies [74].

### 5.2. Colonoscopy

Colonoscopy, the gold standard method for colorectal cancer (CRC) screening, involves the use of a flexible tube with an attached camera to provide a direct view of the colon and rectum. This procedure facilitates both the detection and removal of polyps, making it an effective diagnostic and preventive tool. For individuals at average risk, colonoscopy is recommended every 10 years [75].

Beyond CRC screening, colonoscopy is widely used for diagnosing and managing various colonic conditions, including polyps and inflammatory bowel disease (IBD). The procedure involves inserting a flexible video colonoscope through the rectum and advancing it to the cecum under real-time visualization, allowing for both diagnostic assessment and therapeutic interventions. Proper bowel preparation, typically achieved with polyethylene glycol (PEG) or a split-dose regimen, is crucial for optimal mucosal evaluation, as inadequate preparation can significantly reduce diagnostic accuracy [76].

Sedation practices vary, with options ranging from moderate sedation using midazolam and fentanyl to deep sedation with propofol, which is associated with better patient comfort and higher adenoma detection rates (ADR). To enhance luminal distension and improve lesion detection, insufflation with either air or carbon dioxide is employed. Additionally, high-definition imaging and chromoendoscopy techniques further increase diagnostic precision. A withdrawal time of at least six minutes is recommended to optimize ADR. Various polypectomy techniques, such as cold snare polypectomy for small polyps and endoscopic mucosal resection (EMR) for larger lesions, help reduce CRC risk. When necessary, biopsies are taken for histopathological evaluation [73].

Despite its high diagnostic accuracy, colonoscopy has certain limitations. The procedure carries a small risk of complications, including perforation (<0.1%), post-polypectomy bleeding (0.3–1%), and adverse reactions to sedation. Additionally, incomplete examinations, operator-dependent variability, and the potential for missed lesions highlight the need for quality improvement initiatives. Advancements such as artificial intelligence-assisted polyp detection and robotic colonoscopy are being developed to enhance procedural accuracy and efficiency. Overall, colonoscopy remains a cornerstone of colorectal disease management, with continuous technological advancements improving its safety, effectiveness, and diagnostic capabilities [77].

### 5.3. Flexible Sigmoidoscopy

Flexible sigmoidoscopy is a minimally invasive endoscopic procedure designed to examine the rectum and lower colon, typically extending up to about 60 cm from the anal verge. This test involves inserting a flexible fiber-optic or video sigmoidoscope through the anus to directly visualize the mucosa, facilitating the detection of polyps, inflammation, bleeding, and other abnormalities. Unlike colonoscopy, flexible sigmoidoscopy is usually performed without sedation, and bowel preparation generally consists of enemas or low-volume laxatives. To enhance visualization, insufflation with air or carbon dioxide is used, while biopsy forceps or snares allow for tissue sampling and polyp removal when necessary [78].

Compared to full colonoscopy, flexible sigmoidoscopy offers a lower-risk, quicker alternative for colorectal cancer (CRC) screening, particularly for lesions in the distal colon. Although it does not examine the entire colon, studies have demonstrated its effectiveness in reducing CRC incidence and mortality when performed every five years in average-risk individuals [73].

### 5.4. CT Colonography

CT colonography, also known as virtual colonoscopy, is a non-invasive imaging technique that uses low-dose computed tomography (CT) to generate detailed three-dimensional images of the colon and rectum. This method facilitates the detection of polyps and malignancies without the need for sedation. However, adequate bowel preparation is still required to ensure clear imaging. While CT colonography is a viable alternative to traditional colonoscopy, it may be less effective in identifying smaller lesions, and any abnormalities detected require follow-up colonoscopy for definitive diagnosis [79]. The information presented above is comprehensively summarized in Table 4.

### 5.5. Stool DNA Tests

Stool DNA tests, such as the multitarget stool DNA (mt-sDNA) test (e.g., Cologuard), offer a non-invasive approach to CRC screening by detecting genetic and molecular markers associated with colorectal neoplasia in stool samples. These tests are recommended every three years for average-risk individuals and provide the advantage of requiring no bowel preparation or dietary restrictions. However, while convenient, stool DNA tests have a lower sensitivity for identifying precancerous polyps compared to colonoscopy. Furthermore, positive results necessitate a follow-up colonoscopy to confirm findings and guide further management [80]. Both CT colonography and stool DNA tests expand screening options, particularly for individuals unable or unwilling to undergo traditional colonoscopy. However, each method has limitations that must be considered in clinical decision-making.

## 6. Biosensors as Alternatives to Traditional Diagnostic Methods

Biosensors are analytical tools that integrate a biological sensing component with a physical transducer to identify specific biological or chemical targets. These devices generate a measurable output when they encounter substances such as DNA, proteins, cells, or metabolic compounds. Their use has expanded significantly across various fields, including clinical diagnostics, environmental assessment, food quality control, and biotechnological research [81,82].

**Figure 2:**
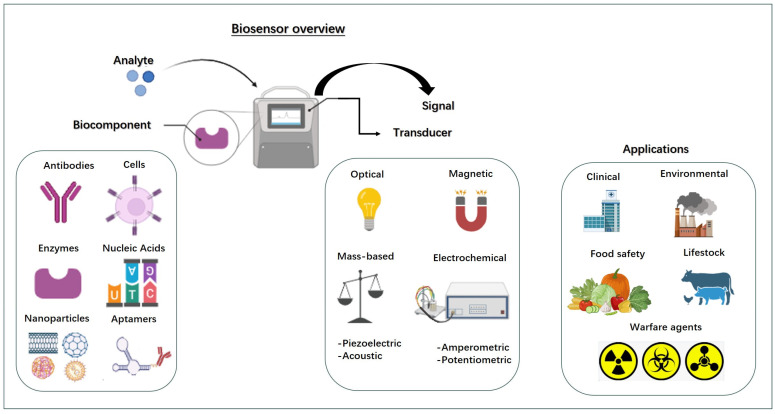
Schematic illustration of biosensor components and applications.

A typical biosensor comprise three essential parts (Figure 2): (i) a biological recognition element, such as enzymes, antibodies, nucleic acids, aptamers, or whole cells that binds specifically to the target analyte, (ii) a transducer that transforms this interaction into a detectable signal and (iii) a processing unit or display system that enhances and presents the signal. Depending on the nature of the signal conversion, biosensors are commonly divided into categories like electrochemical, optical, piezoelectric, thermal, and electronic types [81].

### 6.1. Electrochemical Biosensors

Electrochemical biosensors represent one of the most mature and widely applied classes of analytical platforms in biomedical diagnostics. Their operation relies on converting biochemical recognition events into quantifiable electrical signals, typically involving changes in current, potential, or impedance at the electrode interface. Due to their high sensitivity, cost-effectiveness, and ease of miniaturization, these devices have become indispensable tools for early cancer detection, including colorectal cancer (CRC) [83,84].

#### 6.1.1. Amperometric Biosensors

Amperometric biosensors detect current alterations resulting from oxidation or reduction reactions of electroactive species at the electrode surface. This type of sensor measures the electron transfer rate generated during a redox process, producing a signal proportional to analyte concentration. Amperometric devices are commonly employed in glucose sensors but have recently been adapted for detecting tumor markers relevant to CRC [83]. For instance, polypyrrole-based amperometric biosensors have been developed for the highly sensitive detection of carcinoembryonic antigen (CEA) and methylated *Septin9* DNA, achieving exceptional analytical performance due to the conductive and biocompatible nature of the polymer [85]. Similarly, the integration of gold nanoparticles (AuNPs) and carbon nanotubes (CNTs) onto amperometric electrodes enhances surface area and facilitates electron transfer, leading to improved sensitivity in biomarker quantification [84]. Another [86] study reports the development of a pioneering electrochemical immunoplatform employing gold–silica nanoconjugates (Au@SiO_2_NCs) functionalized with biotinylated antibodies and horseradish peroxidase (HRP) for the ultrasensitive detection of chemokine ligand-12 (CXCL12), a key biomarker in colorectal cancer (CRC) progression. The nanoconjugates act as multifunctional labels, enhancing signal amplification through high enzyme loading and optimized antibody orientation, enabling a detection limit of 22 pg/mL and a fourfold increase in sensitivity over conventional assays. Easily fabricated using commercial materials, the platform accurately quantified CXCL12 in plasma from CRC patients and healthy individuals.

Furthermore, a recent work [87] presents the first electrochemical bioplatform specifically designed to quantify both the total and N6-methyladenosine (m6A)-methylated forms of target microRNAs, exemplified by miRNA let-7a, a key regulator implicated in colorectal cancer (CRC) development and progression. The platform operates through the hybridization of miRNA with a synthetic biotinylated DNA probe, followed by magnetic microcarrier-based capture and selective antibody recognition—either for the DNA/miRNA heterohybrid or for the m6A epimark—using horseradish peroxidase (HRP)-labeled secondary antibodies to enable amperometric detection on disposable electrodes. This integrated, highly sensitive system completes analysis in less than 75 min and successfully differentiates between healthy and cancerous tissues, as well as metastatic potential, demonstrating for the first time the viability of assessing miRNA methylation electrochemically as a novel prognostic and diagnostic approach for CRC.

#### 6.1.2. Potentiometric Biosensors

Potentiometric biosensors measure the potential difference between two electrodes under negligible current flow, typically employing ion-selective or enzyme-modified membranes. These devices are particularly advantageous for their rapid response and low power consumption.

In CRC research, potentiometric transducers have been used to quantify ions or charged biomolecules associated with tumor metabolism. Their miniaturization allows on-chip integration for real-time and point-of-care analysis. Although less frequently applied than amperometric systems, potentiometric sensors show promise for detecting pH or ionic variations linked to tumor microenvironment changes, a characteristic feature of CRC progression [83,88].

Additionally, DPV-based biosensors utilizing T7 endonuclease I for mismatch-specific cleavage and horseradish peroxidase (HRP) for signal amplification have enabled precise detection of *KRAS* mutations, allowing the identification of low-abundance mutant allele [89].

Electrochemical biosensors have proven particularly effective in nucleic acid and miRNA detection, crucial for early CRC diagnosis. Using DNA or RNA aptamers as recognition elements, these devices transduce hybridization or binding events into measurable electrochemical signals [90]. Platforms targeting miR-21 and miR-92a, both closely associated with CRC progression, have demonstrated exceptional sensitivity and reproducibility [91,92]. For instance, a urinary miRNA biosensor employing redox-active methylene blue tags achieved detection limits in the femtomolar range [92].

#### 6.1.3. Impedimetric Biosensors

Impedimetric biosensors monitor changes in the electrical impedance caused by biomolecular binding at the electrode surface. This approach offers a label-free detection mechanism with high specificity and reproducibility [93].

An exemplary application is the label-free impedimetric immunosensor for the early detection of Colorectal Cancer-Specific Protein-2 (CCSP-2). The sensor uses a monoclonal antibody immobilized on a gold electrode, detecting impedance shifts upon antigen–antibody binding in patient serum samples. This platform demonstrated remarkable sensitivity and analytical reproducibility, providing a promising avenue for minimally invasive CRC diagnosis [94].

Another variation, the electrolyte–insulator–semiconductor (EIS) biosensor, enables detection of *KRAS* and *BRAF* mutations with excellent stability and minimal drift, allowing accurate genotyping from clinical samples [95]. Such systems are particularly useful in personalized medicine, guiding targeted therapies for CRC. Another work [96] introduces a label-free electrochemical aptasensor for interleukin-6 (IL-6) built on a glassy carbon electrode sequentially modified with p-aminobenzoic acid, p-aminothiophenol, and gold nanoparticles, where a 3′-thiolated IL-6 aptamer is immobilized via Au–S bonds. The platform transduces IL-6 binding by electrochemical impedance spectroscopy, delivering a wide linear range (5 pg mL^−1^–100 ng mL^−1^), ultra-low detection limit (~1.6 pg mL^−1^), and high selectivity against common interferents, thanks to AuNP-enabled surface area and stable aptamer anchoring. Clinically, it quantified IL-6 in serum from colorectal cancer patients with recoveries validated by CLIA, operating near physiological cut-offs (≈4–6 pg mL^−1^) and demonstrating promise for sensitive CRC screening and monitoring.

Finally, a label-free impedimetric biosensor combining Ti_3_C_2_Tₓ MXene and an imprinted ortho-phenylenediamine (o-PD) polymer for the rapid and sensitive detection of carcinoembryonic antigen (CEA) was also reported. The MXene-modified disposable silver electrode enhances conductivity, surface area, and protein immobilization, while the molecularly imprinted polymer ensures selective CEA recognition. Demonstrating a detection limit of 9.41 ng/mL, a linear range of 10–100 ng/mL, and high reproducibility, this cost-effective, 10 min assay shows excellent agreement with immunoassay results, positioning it as a powerful point-of-care tool for cancer diagnosis and treatment monitoring.

#### 6.1.4. Novel Multiplexed and Integrated Electrochemical Biosensing Platforms

Recent developments focus on multiplexed systems capable of detecting several CRC-related biomarkers simultaneously. Platforms designed to measure multiple epigenetic modifications (5-methylcytosine, 5-hydroxymethylcytosine, DNA N^6^-methyladenine, RNA N^6^-methyladenosine) allow accurate differentiation between tumor and normal tissues [89].

An innovative electrochemical immunosensing [97] platform was designed for the simultaneous detection of eight colorectal cancer (CRC)-associated autoantibodies, enabling both easy- and difficult-to-express tumor-associated antigens (TAAs) to be integrated into a single diagnostic device. The system employs HaloTag fusion proteins produced through an in vitro transcription/translation method, covalently immobilized onto magnetic microbeads that capture disease-specific autoantibodies from plasma samples. By coupling this biorecognition process with amperometric detection at disposable screen-printed carbon electrodes, the platform demonstrated high sensitivity, reproducibility, and clear discrimination between CRC patients, high-risk individuals, and healthy or non-CRC controls. In addition, another study presented [98] a simple and highly sensitive dual electrochemical immunoplatform for the simultaneous detection of mucin 1 (MUC1) and mucin 16 (MUC16), two biomarkers associated with colorectal cancer (CRC) progression and minimal residual disease. The platform employs functionalized magnetic beads and amperometric detection at dual screen-printed carbon electrodes, achieving remarkable detection limits of 50 pg/mL for MUC1 and 1.81 pg/mL for MUC16 without the need for nanomaterials.

Moreover, another study [99] presents the first electrochemical bioplatforms capable of individually or simultaneously detecting all four cytosine methylation marks—5mC, 5hmC, 5fC, and 5caC—central to the DNA methylation-demethylation cycle and pivotal in cancer epigenetics. Using direct competitive immunoassays on magnetic microbeads and amperometric detection at disposable electrodes, the platforms achieved picomolar sensitivity and enabled rapid, selective, and multiplexed analysis of these epimarks. Applied to genomic DNA from paired healthy and colorectal tumor tissues, the technology revealed distinct methylation patterns and inter-epimark correlations, offering groundbreaking insight into tumor biology and potential applications for precise cancer diagnosis and surgical outcome assessment.

Integration of electrochemical sensors with microfluidic chips and AI-assisted data analysis further enhances detection accuracy and allows portable, automated operation. An electrochemiluminescence biosensor combining Ag_3_PO_4_ nanoparticles with MoS_2_ nanosheets has achieved sensitive detection of *KRAS* genes in both tumor and adjacent normal tissue, offering a valuable complement to liquid biopsy for early CRC screening [89,100].

Collectively, electrochemical biosensors integrate molecular, genetic, and clinical insights into a unified, non-invasive diagnostic platform for colorectal cancer. Their adaptability, low cost, and analytical precision make them indispensable for early detection, disease monitoring, and therapeutic assessment. Ongoing integration with nanotechnology, microfluidics, and artificial intelligence promises to transform them into next-generation point-of-care systems, capable of real-time and multiplexed CRC diagnostics [94,95,101].

### 6.2. Optical Biosensors

Optical biosensors have emerged as powerful analytical tools in biomedical research because of their exceptional sensitivity, real-time response, and potential for miniaturization. These devices detect subtle variations in optical properties that occur when a target molecule interacts with a specific biological recognition element. A typical optical biosensor includes a bioreceptor, such as an antibody, enzyme, aptamer, or nucleic acid sequence, which provides molecular specificity; a transducer that converts this recognition event into an optical signal; and a detection system that quantifies the resulting signal [102,103].

Depending on the sensing mechanism, the optical response may involve changes in fluorescence, luminescence, absorbance, refractive index, or surface plasmon resonance (SPR). In fluorescence-based biosensors, target binding may lead to quenching or enhancement of emitted light. At the same time, in SPR systems, adsorption of biomolecules at a metal–dielectric interface causes a measurable shift in the resonance wavelength. These fundamental principles enable label-free, highly sensitive, and rapid detection of analytes [104]. Owing to their versatility and compatibility with micro- and nanostructured materials, optical biosensors have become particularly relevant for the early detection of colorectal cancer (CRC) biomarkers, where accurate and rapid quantification of disease-related molecules can significantly improve diagnostic outcomes [105].

#### 6.2.1. Fluorescence-Based Biosensors

Fluorescence detection remains one of the most widely used optical strategies for CRC biomarker analysis, offering excellent sensitivity and ease of integration into portable formats. A variety of fluorescence-based lateral flow immunoassays (LFIAs) and nanomaterial-assisted platforms have been reported for the detection of key CRC markers. Li et al. have presented several examples, including a quantum-dot-based LFIA employing CdSe@ZnS nanocrystals, which achieved a detection limit of approximately 2 pg/mL for interleukin-6 (IL-6) [106]. In another study, europium(III)-chelate-doped nanoparticles (EuNPs) served as fluorescent reporters for IL-6 quantification, reaching a limit of detection of 0.37 pg/mL. Comparable precision was observed when results were benchmarked against standard chemiluminescence assays. Similarly, a colorimetric LFIA using Fe_3_O_4_–CNT composites for CA19-9 detection achieved a detection limit near 1.75 U/mL, demonstrating the potential of magnetic nanomaterials to enhance signal clarity and specificity in complex biological matrices.

Beyond serum-based testing, optical biosensing has also been applied to tissue diagnostics. Wu et al. designed a fluorescence sensor array built from nanoscale metal–organic frameworks (NMOFs) that could distinguish normal, adenomatous, and cancerous colorectal tissues [107]. The array employed three structurally distinct NMOFs—copper-, iron-, and zirconium-based—as quenchers for fluorescently labeled DNA reporters. When exposed to tissue extracts, each framework generated a unique fluorescence pattern. Linear discriminant analysis of these optical fingerprints allowed accurate classification of tissue types, achieving diagnostic accuracy comparable to histopathological assessment. This approach demonstrates how optical biosensing can capture global proteomic differences, offering a rapid, label-free adjunct to conventional pathology.

#### 6.2.2. Plasmonic and Surface Plasmon Resonance (SPR) Biosensors

Plasmonic biosensors, relying on surface plasmon resonance (SPR) or localized surface plasmon resonance (LSPR), are distinguished by their ability to monitor biomolecular interactions in real time without labeling. Ahmadzadeh-Raji et al. [108] presented an optically transparent aptamer-based biosensor that combined electrodeposited gold nanoparticles (AuNPs) with an indium tin oxide (ITO) substrate. This nanostructured ITO/AuNP layer not only offered high electrical conductivity and optical transmittance (>80%) but also served as an efficient platform for aptamer immobilization via thiol–gold bonding. Upon target binding, distinct shifts in optical absorbance and impedance confirmed successful dual-mode optical–electrochemical sensing. The system displayed a linear detection range in the picomolar-to-nanomolar regime with rapid response times under five minutes.

Additional developments in this field include aptamer-based SPR biosensors for proteins associated with CRC, such as heterogeneous nuclear ribonucleoprotein A1 (hnRNP A1) and carcinoembryonic antigen (CEA) [106]. An SPR device using a CEA-specific aptamer achieved a detection limit of 17.8 pg/mL, while another targeting hnRNP A1 demonstrated sensitivity down to 0.22 nM in plasma samples. These findings underline the precision and reproducibility of plasmonic detection, especially when paired with high-affinity binding molecules.

A complementary advancement is the use of Affimers, engineered small protein scaffolds that mimic antibody binding. Shamsuddin et al. identified three Affimer binders specific to CEA and demonstrated their strong and selective binding to both native and deglycosylated CEA forms. Due to their compact structure and stability, Affimers can be densely immobilized on sensor surfaces, resulting in enhanced signal uniformity. Their adaptability and reproducibility make them promising alternatives to traditional antibodies for the next generation of optical biosensors targeting colorectal cancer biomarkers [109].

#### 6.2.3. Surface-Enhanced Raman Scattering (SERS) Biosensors

Surface-enhanced Raman scattering (SERS) biosensors amplify molecular vibrational signals by exploiting the strong electromagnetic fields generated at metallic nanostructures. These sensors can achieve single-molecule sensitivity while retaining the molecular fingerprint information characteristic of Raman spectroscopy. Li et al. [106] reported a highly sensitive SERS-based immunosensor for CEA detection that utilized Fe_3_O_4_@Au/Ti_3_C_2_Tx MXene composites as magnetic capture substrates and MoS_2_–Au nanoflower hybrids as Raman tags. Using 4-mercaptobenzoic acid as a reporter molecule, the device provided a detection range from 0.1 pg/mL to 100 ng/mL, with a limit of detection as low as 0.033 pg/mL.

Such systems highlight the remarkable analytical power of SERS biosensing, capable of detecting extremely low concentrations of CRC biomarkers in biological fluids. The main limitations involve the complexity of nanostructured substrate fabrication and potential spectral variability [110]. Nevertheless, ongoing advances in reproducible nanofabrication are rapidly improving the reliability of this method for clinical applications.

#### 6.2.4. Optical Imaging and Endoscopic Biosensing

Recent innovations have extended the concept of optical biosensing to real-time imaging systems capable of operating inside the gastrointestinal tract. Fontana et al. [111] designed a magnetically navigable spherical capsule equipped with a miniaturized CMOS camera and concentric LED illumination. The capsule, measuring 26 mm in diameter and weighing only 12.7 g, transmitted 320 × 320 pixel images at 1.5 frames per second via Bluetooth. Data were processed in real time by an onboard FPGA chip, which also performed image compression, while wireless inductive charging enabled reusability. This device demonstrated high-quality mucosal visualization and represents a significant step toward minimally invasive optical diagnostics for CRC.

In a related development, Gerald et al. [112] created a soft optical blood sensor designed to detect minute traces of bleeding during colonoscopy. The sensor integrates dual-wavelength LEDs and a photodiode within a flexible PDMS matrix, operating via diffuse reflectance photometry. It distinguishes hemoglobin and oxyhemoglobin based on their absorption spectra at 540–560 nm and 650–700 nm, respectively. Ex vivo testing on porcine colon tissue revealed a linear correlation between optical reflectance and blood concentration, with detection sensitivity down to 0.1% blood volume. The sensor’s conformal, soft design minimizes tissue trauma and ensures stable optical coupling, making it compatible with robotic or capsule-based endoscopic systems.

Together, these imaging innovations exemplify the integration of optical biosensing principles into active diagnostic platforms, enabling both visualization and quantitative analysis of physiological changes associated with colorectal lesions [113].

The growing family of optical biosensors, including fluorescence-based assays, plasmonic and SPR systems, SERS platforms, and imaging devices, offers a broad spectrum of analytical capabilities for CRC detection [114]. Each modality provides distinct advantages: fluorescence assays deliver rapid and low-cost quantification suitable for point-of-care testing; plasmonic and SPR sensors allow label-free, real-time monitoring with sub-nanomolar precision; and SERS achieves unparalleled sensitivity with molecular specificity. Imaging-based optical sensors extend these principles into in vivo applications, allowing clinicians to detect and visualize lesions in real time [115].

Given the multifactorial nature of colorectal cancer, reliance on a single biomarker remains insufficient for reliable diagnosis. Future efforts are expected to focus on multiplexed optical biosensors capable of simultaneously detecting multiple molecular targets such as CEA, CA19-9, IL-6, IL-8, VEGF, and EGFR. Combining these detection platforms with microfluidic integration, AI-assisted data interpretation, and soft photonic materials could enable truly portable, autonomous systems for early CRC screening and ongoing disease monitoring.

### 6.3. Piezoelectric Biosensors

Piezoelectric biosensors operate by detecting minute mass changes at the sensor surface, which affect the resonance frequency of a piezoelectric crystal—typically quartz. This principle enables highly sensitive, label-free detection of molecular interactions, such as antigen–antibody binding or nucleic acid hybridization, real-time response, and compatibility with miniaturized and portable platforms [116]. Despite their sensitivity, these devices are susceptible to environmental variables like vibration and temperature, which can affect performance [117]. By embedding such sensors into endoscopic tips or flexible catheters, clinicians can obtain real-time feedback on tissue elasticity and surface texture, complementing visual imaging for improved lesion characterization. New approaches focused on integrating piezoelectric tactile sensors into endoscopic instruments to enhance diagnostic capabilities [118,119]. These systems successfully distinguished between soft and hard tissue regions, allowing for enhanced polyp classification accuracy compared to standard visual methods alone. This approach highlights the potential of tactile sensing to reduce misdiagnosis rates and improve the sensitivity of early cancer detection.

An inflatable vision-based tactile sensing balloon (VTSB) designed to enhance colorectal cancer (CRC) polyp detection during colonoscopy has been proposed by Kara et al. (2023) [119]. The VTSB integrates with standard colonoscopes to provide radiation-free, high-resolution texture and morphology mapping of polyps, demonstrating strong potential to improve early CRC diagnosis [119]. In another study, an innovative quartz crystal microbalance (QCM) immunosensor has been reported for the detection of carcinoembryonic antigen (CEA) in serum from colorectal cancer patients, utilizing an L-cysteine-modified gold electrode. Monoclonal anti-CEA capture antibodies were immobilized and employed in a sandwich-type assay with horseradish peroxidase (HRP) nanoparticle enhancers that catalyze 4-chloro-1-naphthol oxidation, producing an insoluble precipitate upon CEA detection. The resulting precipitation-induced mass changes on the QCM probe enabled sensitive and efficient frequency-based quantification (Chi et al., 2020) [120]. Following a similar concept, graphene oxide–gold nanoparticles (GO–AuNPs) were synthesized in situ, on a QCM electrode surface, and monoclonal anti-CEA antibodies (from mouse) were covalently immobilized on this layer as bioreceptors. The biosensor effectively recognized and bound to CEA biomolecules (Jandas et al., 2020) [121]. A QCM has been applied for the sensitive detection of the colorectal cancer biomarker miR-221miRNA, presenting a detection limit at 6.9 fM, employing a biotin-modified DNA probe and a streptavidin@metal–organic framework complex for recognition elements and signal amplification (Ma et al., 2020) [122].

Furthermore, another subcategory of piezoelectric sensors, surface acoustic wave (SAW) sensors [123,124], which operate based on the propagation of acoustic waves along piezoelectric substrates, have demonstrated significant potential for cancer biomarker detection due to their strong sensitivity to alterations in mass loading and viscoelastic properties at the sensor surface [124,125,126]. SAW sensors are highly versatile, enabling the detection of a broad range of biomarkers, from volatile organic compounds (VOCs) to larger biomolecules such as proteins and DNA.

For gastrointestinal cancer detection, a Love wave sensor functionalized with a molecularly imprinted polymer (MIP) using adenosine monophosphate (AMP) as a model target molecule has been proposed [127]. Love wave devices are based on their guided nature in a thin layer, which ensures a high confinement of acoustic energy near the sensing surface. By comparing responses from MIP-coated, NIP-coated, and bare sensors, the study provides insights into the thin-film morphology, porosity, and imprinting effects. The results demonstrate good reproducibility of the polymer films and show that MIP coatings enhance sensor sensitivity by a factor of 3–4 relative to NIP-coated or uncoated devices. The combined use of highly selective MIP layers and sensitive Love wave devices proves effective, and future work will focus on detecting nucleoside colorectal cancer biomarkers using newly synthesized MIPs integrated with microfluidic systems. Moreover, a sensitive and rapid immunofluorescence sensor combining metal-enhanced fluorescence with Rayleigh surface acoustic waves was proposed for detecting carcinoembryonic antigen (CEA) [128]. Silver nanocubes significantly amplify fluorescence signals, lowering the detection limit to below 1 ng/mL, while acoustic streaming improves mixing, removes nonspecific binding, and greatly reduces incubation times. Together, these effects enable fast and reliable CEA detection at clinically relevant concentrations and identify key parameters for optimizing sensor performance.

### 6.4. Electronic (FET-Based) Biosensors

Electronic biosensors, particularly those based on field-effect transistors (FETs), detect changes in surface electrical properties—such as charge distribution—that occur when target analytes bind to the transistor’s gate area. Owing to their ability to offer real-time, label-free detection and their suitability for miniaturization and integration into lab-on-a-chip systems, FET-based biosensors show significant potential for point-of-care diagnostics. They are increasingly being used in the detection of cancer biomarkers and in identifying infectious agents [129]. An electrical double layer (EDL)-gated AlGaN/GaN high electron mobility transistor (HEMT) biosensor array was developed and characterized to monitor transmembrane potential changes in cells. Designed for detecting and counting circulating tumor cells (CTCs) in colorectal cancer, the platform enables real-time study of cellular bioelectric signals under physiological conditions without complex automation. The sensor response correlates with membrane potential variations in captured cells, and its performance was evaluated under changing ion concentrations and cadmium-induced ion channel blocking. This EDL FET biosensor shows promise as an electrophysiological probe and a rapid point-of-care diagnostic tool for disease screening [130]. An electric-field effect colorectal sensor (E-FECS), designed as a dual-gate ion-sensitive field-effect transistor with a nanostructured surface, was developed to directly quantify Colon cancer secreted protein-2 (CCSP-2) from patient blood samples. The sensor’s performance was verified in 7 controls and 7 CRC samples and clinically validated across 30 controls, 30 advanced adenomas, and 81 CRC cases. The E-FECS system effectively detected CCSP-2 across various disease stages, including early-stage CRC, highlighting its potential as a reliable diagnostic tool for blood-based CRC screening [131]. Pan et Liao developed a YbTixOy-based electrolyte–insulator–semiconductor (EIS) biosensor for detecting KRAS and BRAF gene mutations in colorectal cancer patients [132]. The EIS field-effect transistor (FET) structure allows direct interaction with biological fluids, offering high sensitivity to variations in ion concentration and pH. This design enables label-free, real-time genetic detection, providing a simpler and faster alternative to conventional molecular assays. The YbTixOy EIS biosensor demonstrates strong potential for clinical applications in colorectal cancer diagnosis and personalized treatment monitoring. A portable detection platform based on a planar-gate graphene field-effect transistor functionalized with a polydopamine self-assembled film (PDA-GFET) was developed for rapid colon cancer identification [89]. The biosensor detects the EpCAM protein, expressed on colon cancer-derived exosomes, directly from clinical samples within 10 min. The PDA coating enhances the functionalization area and minimizes non-specific adsorption, achieving an ultralow detection limit of 112 particles/mL. Clinical testing revealed significant differences between healthy and cancer patient samples (*p* < 0.001). The PDA-GFET platform demonstrates strong potential as a rapid, sensitive, and non-invasive tool for early colon cancer diagnosis.

### 6.5. Nanomaterials for Sensor-Based Applications

Nanotechnology offers a diverse range of nanomaterials with significant diagnostic and therapeutic potential. Materials and formulations such as carbon nanotubes, dendrimers, liposomes, silica nanoparticles, gold nanoparticles, graphene oxide, metal–organic frameworks, core–shell polymeric nanostructures, and nanoemulsion systems have been explored for targeted anticancer drug delivery and diagnostic applications in CRC [133]. These materials increase the electrode’s surface area, improve conductivity, and provide sites for covalent attachment of biomolecules [134]. Recent studies demonstrated graphene oxide nanocomposite-based electrochemical sensors capable of detecting CEA at ultralow concentrations, relevant to CRC diagnosis [135]. Another example involves MXene/Au hybrid systems, which enhance redox activity and reduce background noise, thereby improving detection precision for protein and nucleic acid biomarkers [136]. Such nano-engineered electrodes enable the measurement of biomarkers, including VEGF, miRNA-21, and ATP, facilitating high-resolution molecular profiling of CRC progression [84].

Nonetheless, conducting polymers, especially polypyrrole (PPy) and polyaniline (PANI), have been extensively explored as transducer materials for electrochemical biosensors due to their conductivity, stability, and tunable surface chemistry. PPy-based platforms have demonstrated significant potential for CRC biomarker detection, enabling simultaneous monitoring of CEA and methylated DNA biomarkers with high precision [85]. The combination of PPy matrices with nanostructured metals or oxides enhances the electrode’s electrocatalytic activity and provides more binding sites for biomolecular immobilization. These hybrid architectures support reproducible and low-noise signal transduction, essential for reliable CRC diagnostics [90].

Nanomaterial-based sensors are able to identify miRNA and DNA mutations (such as those in the KRAS and BRAF genes), even when using small sample volumes, which facilitates minimally invasive liquid biopsies [137]. MicroRNA-21 (miR-21), a well-established oncogenic regulator, stands out as a key molecular target in colorectal cancer (CRC). A multivariate-gated catalytic hairpin assembly (CHA) nanosensor was introduced by Zhang et al. [138], which amplifies imaging of miR-21 in human CRC tissues. By combining endogenous glutathione and exogenous near-infrared (NIR) gating with CHA probes, this nanosensor enhanced signal intensity and reduced background noise, resulting in a 1.6-fold increase in signal-to-background ratio compared to conventional CHA techniques. Similarly, an NIR-II-Luminescence (NIR-II-L, 1500–1900 nm) sensor for CRC was developed, based on the synergizing of DNAzyme-driven signal amplification with a lanthanide–dye hybrid system [139]. This method enabled non-invasive visualization of orthotopic colorectal cancer via rectal delivery, achieving an exceptionally low detection limit of 1.26 pM and allowing diagnosis up to two weeks earlier than standard histology. Extending miRNA detection capabilities, a Mn^2+^-modified black phosphorus (BP@Mn^2+^/DNA) hybrid nanosensor has been proposed that detects exosomal miRNAs and exosomes from CRC cells [140]. This ultra-thin two-dimensional sensor could autonomously penetrate exosome membranes and distinguish CRC-derived exosomes from those of healthy intestinal epithelial cells based on miRNA profiles. Functionalization with an epithelial cell adhesion molecule (EpCAM) aptamer allowed differentiation between plasma exosomes from CRC patients and healthy individuals, showcasing its dual function in molecular and vesicular biomarker detection.

Beyond miRNAs, DNA methylation profiling has emerged as a powerful diagnostic approach. By employing a single-molecule Natural Killer cell DNA analysis with an ultrasensitive Nickel 3D nanosensor combined with Raman spectroscopy, researchers identified distinct CRC-specific methylation patterns in NK cells by comparing those associated with CRC to healthy counterparts [141]. These epigenetic markers were then integrated into a machine learning framework to develop a predictive model for CRC diagnosis. Luo et al. proposed a nanoparticle-coupling and site-specific oxidation strategy for quantitative DNA methylation assessment without requiring amplification. Their nanosensor demonstrated ultra-high sensitivity with a detection limit of 3.2 × 10^−16^ M and could detect methylation levels as low as 0.001%, outperforming the clinically approved Septin 9 assay [142]. This capability enables precise monitoring of CRC progression and therapeutic outcomes. A genosensor has been proposed for detecting the methylation status of a BMP3 gene fragment, an important epigenetic biomarker used in the FDA-approved sDNA test for colorectal cancer screening. Signal amplification and increased sensitivity were achieved using polyethyleneimine-stabilized silver nanoparticles (PEI-AgNPs) as non-specific nanolabels. The genosensor showed a linear response across a target concentration range from 1 fM to 100 nM, with a detection limit of 1 fM, allowing accurate differentiation between methylated and unmethylated sequences [143].

Nanosensors have also targeted protease activity, a hallmark of tumor progression. Holt et al. developed a nanoparticle–substrate protease sensor for matrix metalloproteinase 9 (MMP9), where peptide substrates cleaved by MMP9 release fluorescent markers into urine for non-invasive quantification [144]. Building on this, Van Heest et al. an activity-based nanosensors with DNA barcodes released by tumor-associated proteases was introduced, detected via CRISPR nucleases for multiplexed urine-based protease activity readouts [145]. This field was further advanced by the creation of a multimodal nanosensor incorporating tumor-targeting via acidosis, protease responsiveness, and optional PET imaging [146]. This platform enabled concurrent urinary protease detection and pH-dependent PET scans for comprehensive, longitudinal tumor monitoring and therapeutic evaluation.

Recent advances display the integration of nanosensing with microbiome-related biomarkers, opening novel diagnostic possibilities for CRC. Feng et al. developed a colorimetric aptasensor targeting Parvimonas micra, a bacterium enriched in CRC tissues and stools. Using whole-bacterium SELEX to select high-affinity aptamers linked to Au@Fe_3_O_4_ nanoparticles, this system sensitively and linearly detected P. micra at 11 CFU/mL and successfully distinguished CRC patient samples from healthy controls, emphasizing the diagnostic relevance of microbial dysbiosis [147].

### 6.6. VOC-Based Biosensors

VOC-based biosensors aim at detecting metabolic by-products of tumorigenic processes in breath or stool samples, offering a new and patient-friendly approach to CRC screening [85,148]. Recent studies have investigated the use of volatile organic compounds (VOCs) analysis as a diagnostic tool for cancer detection, with a special focus on colorectal cancer (CRC). Breath sampling combined with sensor platforms like gold nanoparticle (GNP) and metal oxide (MOX) sensors has shown promising results. For example, one study assessed a tabletop breath analyzer using MOX sensors and reported strong diagnostic accuracy when the data were analyzed via supervised machine learning models [149]. Further research has explored nanosensors based on transition metal dichalcogenides (TMDs), particularly tungsten diselenide (WSe_2_) and tungsten ditelluride (WTe_2_) monolayers, which demonstrated high sensitivity to CRC-related VOCs such as benzaldehyde, indole, and propan-2-ol, detecting concentrations from ppm to ppt levels. These results highlight the potential of nanoscale sensors for early CRC diagnosis [150].

Chemoresistive nanosensors have also been utilized to monitor VOCs from blood samples of CRC patients during a one-year follow-up. A device integrating four metal oxide sensors successfully distinguished samples at different treatment phases, achieving 93% sensitivity and 82% specificity when differentiating pre-surgery from long-term post-surgery samples, indicating utility in patient monitoring and relapse detection [151]. A later study expanded this approach to a three-year follow-up, exploring gender-related patterns and reinforcing the feasibility of nanostructured gas sensors for detecting CRC relapse [101].

Additional research employed portable electronic nose (e-nose) systems with multiple metal oxide sensors to analyze VOCs in biological fluids. For instance, the PEN3 e-nose combined with gas chromatography–time-of-flight mass spectrometry (GC-TOF-MS) characterized urinary metabolites in CRC patients [152]. Similarly, urine samples assessed by metal oxide gas sensors coupled with solid-phase microextraction were analyzed using a radial basis function neural network trained to distinguish CRC patients from healthy controls [153]. Another study focused on sweat VOCs for CRC detection, utilizing GC-MS alongside a custom-developed e-nose equipped with 14 gas sensors. The analysis of 136 sweat samples from 68 participants demonstrated the system’s capability to identify VOC patterns indicative of CRC [154]. Collectively, these studies highlight the growing potential of VOC-based diagnostics using advanced sensor technologies and machine learning for the non-invasive detection and monitoring of colorectal cancer.

### 6.7. Microfluidic and Lab-on-a-Chip Biosensors

Recent advances in biosensor miniaturization have accelerated the development of lab-on-chip (LoC) platforms for cancer diagnostics. Microfluidic and lab-on-a-chip biosensors offer compact platforms that integrate sample preparation and analysis, efficiently isolating and detecting targets such as CTCs, exosomes, and nucleic acids directly from whole blood. These systems are especially promising for point-of-care CRC diagnostics when combined with technologies like CRISPR, isothermal amplification, or nanoparticle-based detection [155,156]. Yapeter et al. have demonstrated the feasibility of detecting the BRAF p.V600E mutation, a key colorectal cancer (CRC) biomarker, using loop-mediated isothermal amplification (LAMP) reactions integrated with ion-sensitive field-effect transistor (ISFET) sensors [157]. The lab-on-chip (LoC) system demonstrated reliable performance in detecting cancer-associated mutations and shows potential for adaptation to multi-reaction sensing. In particular, the LAMP reactions performed on the LoC device showed allele-specific detection with results comparable to those from a qPCR machine. The system achieved a limit of detection of 500 copies/μL and a time-to-positive under 15 min, confirming its potential for rapid, on-chip genetic analysis in CRC diagnostics. A portable lateral displacement patterned, pump-free (LP) microfluidic chip incorporating CoPt_3_ nanozyme probes was developed for CCSC capture and detection in blood and fecal samples. The system combines magnetic separation with colorimetric readout via peroxidase-mimicking activity, enabling visual and smartphone-based quantification with a detection limit as low as 3 cells/mL. Preliminary clinical studies demonstrated its potential for predicting CRC progression and poor prognosis [158]. A related approach integrated a biocompatible microelectrode with a wireless microfluidic chip to enable real-time monitoring of hydrogen sulfide (H_2_S) and hydrogen peroxide (H_2_O_2_) secretion from living cells. The device distinguished cancerous from normal cells and evaluated chemotherapeutic efficacy through simultaneous detection of both molecules at micromolar levels [159]. Cell-free nucleic acids (cfNAs) have also emerged as promising biomarkers. A dimethyl dithiobispropionimidate (DTBP)-based microchannel platform achieved rapid cfNA capture within 15 min, enabling detection of KRAS and BRAF mutations in plasma and tissue samples from CRC patients. The method correlated highly with whole-genome sequencing results and could be integrated with various downstream detection techniques, offering a simple and low-cost approach for circulating tumor DNA analysis [160].

### 6.8. Integration with Liquid Biopsy

The integration of biosensor technology with liquid biopsy has opened new avenues for non-invasive cancer diagnostics, particularly in the early detection and monitoring of colorectal cancer (CRC). Electrochemical biosensors have been tailored to identify circulating biomarkers such as ctDNA, miRNAs, and exosomal components in body fluids like plasma and serum. Advanced nanostructured materials such as gold nanoparticles and graphene-modified electrodes significantly enhance detection sensitivity, enabling the identification of low-abundance biomarkers, including methylated SEPT9 DNA and miR-21 in CRC patients [161]. Label-free electrochemical systems further allow for the direct detection of ctDNA hybridization via impedance or current measurements. Optical biosensors, including those based on surface plasmon resonance (SPR) and fluorescence, have demonstrated high specificity in detecting exosomal surface proteins such as CD63 and EpCAM, as well as oncogenic mutations like KRAS and BRAF in ctDNA [162].

Recent research has focused on creating advanced biosensing platforms for efficient detection and characterization of exosomes and EVs. Microfluidic technologies are being developed to replace conventional isolation methods, offering integrated detection with minimal sample volumes. For example, a hollow-fiber flow field-flow fractionation system combined with a luminescence immunoassay successfully isolated and analyzed exosomes for colon cancer diagnosis [163]. Additionally, ultrasensitive mid-infrared plasmonic nanoantenna arrays have been designed for EV detection and molecular profiling, linking EV protein signatures to tumor malignancy [164]. A lateral flow immunoassay targeting the CD147 antigen on circulating EVs showed promise as a rapid, point-of-care screening tool for CRC [165]. Moreover, a label-free method using nanoengineered 3D surface-enhanced Raman spectroscopy (SERS) sensors enabled simultaneous detection of cancer stem cell–derived EVs, demonstrating high diagnostic accuracy across several cancers, including CRC [166]. Together, these innovations indicate that EV-based biosensing technologies have the potential to transform non-invasive cancer diagnosis and monitoring, but despite these advances, challenges persist in the translation of biosensor technologies into clinical practice. Challenges, including the complexity of matrices, reproducibility, and cross-platform standardization, need to be tackled prior to broad implementation.

### 6.9. Advantages and Limitations of Biosensors in CRC Diagnosis

Biosensors have become potent instruments for the early identification of colorectal cancer (CRC), providing high sensitivity and specificity along with quick analysis. These devices can detect a range of CRC-related biomarkers—including genetic mutations (e.g., KRAS, BRAF), epigenetic changes (e.g., methylated SEPT9), protein markers like carcinoembryonic antigen (CEA), microRNAs (miRNAs), and volatile organic compounds (VOCs)—often from minimally invasive samples such as blood, serum, feces, or breath [80,88,125,167,168,169]. Electrochemical biosensors represent a major advancement, employing signal amplification methods and nanomaterials like graphene, polypyrrole and quantum dots to achieve low detection limits and facilitate multiplex detection [88,91,170]. Furthermore, nanosensors targeting miRNA signatures have demonstrated significant potential in identifying early molecular changes with high precision [91]. Biosensors capable of detecting VOCs provide a novel approach to identifying unique metabolic signatures generated by cancer cells, thus enabling non-invasive breath analysis [80].

**Figure 3:**
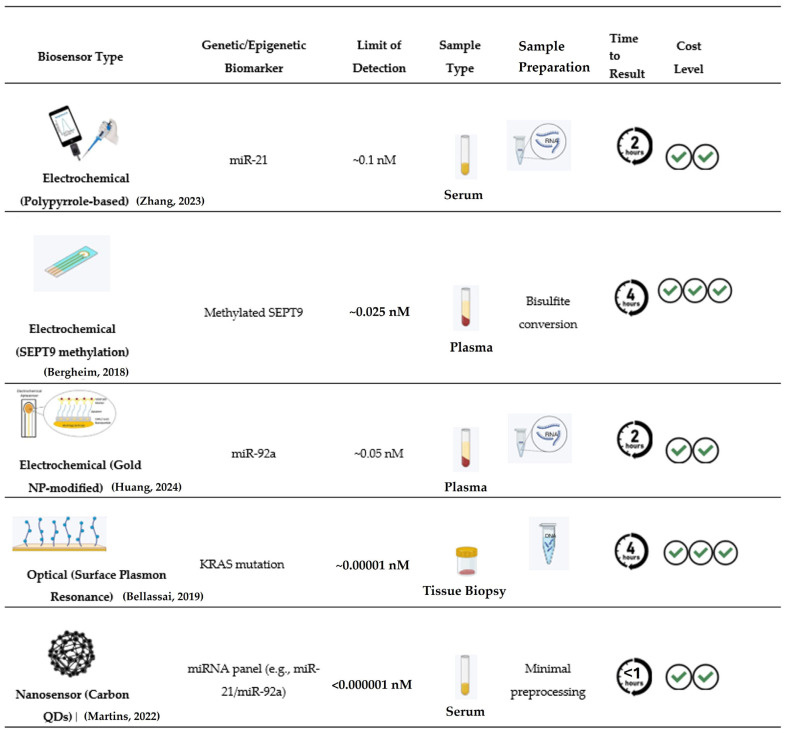
Comparison of CRC biosensors’ performance [85,125,168,169,170].

The appeal of biosensors is further enhanced by their portability, low cost, and potential for point-of-care use (as they are presented in Figure 3), particularly in resource-poor environments where traditional approaches such as colonoscopy are constrained by financial and infrastructural factors [137]. Even with these advantages, difficulties persist. Issues with reproducibility, intricate sample matrices, absence of standardization, and the necessity for thorough clinical validation continue to limit their current clinical use [91].

As aforementioned, biosensors represent a powerful class of analytical tools that combine biological specificity with physicochemical detection. Each category offers distinct advantages and limitations depending on the intended application. The continued development and integration of nanomaterials, microfluidics, and digital platforms are expected to further enhance the performance, affordability, and accessibility of biosensor technologies in the near future. One of the fastest-growing fields for biosensor application is cancer detection, designed to analyze biomarkers present in body fluids such as blood, plasma, serum, saliva, or urine, without the need for invasive tissue biopsies, known as liquid biopsy.

In the context of cancer, this approach focuses on identifying a variety of biomarkers that reflect the presence and progression of tumors. Key targets include circulating tumor DNA (ctDNA), circulating tumor cells (CTCs), microRNAs (miRNAs), as well as exosomes, extracellular vesicles, specific proteins, and metabolites. These components provide valuable insights into tumor biology and have emerged as promising tools for early diagnosis, monitoring treatment response, and detecting recurrence.

## 7. Biosensors in CRC Therapeutic Drug Monitoring (TDM)

Effective cancer therapy requires not only precise medication but also the ability to track the effectiveness of those treatments in real time. Current monitoring tools, such as MRI, CT scans, and biopsies, offer delayed and indirect insights into therapeutic response. These techniques are heavily dependent on capturing anatomical changes rather than early molecular signals, making it difficult to detect whether a drug is losing effectiveness, whether resistance is emerging, or whether dosing needs adjustment. As a result, treatment decisions often occur after the cancer has already adapted. On the other hand, laboratory tests can provide meaningful data but display limitations in sensitivity, especially for precancerous polyps and early-stage tumors, sample instability, lack of automation and may be invasive [55,171].

Biosensors offer a breakthrough approach in clinical use, especially in oncology, due to their ability to provide continuous monitoring during chemotherapy, immunotherapy, and targeted molecular treatments. These minimally invasive systems provide continuous, high-sensitivity measurement of circulating chemotherapeutic agents, tumor-derived nucleic acids (ctDNA, miRNA), immune checkpoint proteins, and metabolic markers. By delivering molecular-level data at the point of care, they make it possible to evaluate whether a therapy is effective in real time. Moreover, these biosensors can be incorporated into portable, wearable, or implantable devices as they can be integrated with microfluidics, wireless communication, advanced materials, and AI-driven analytics. This enables clinicians to adjust dosing, detect resistance mechanisms early, and intervene before treatment failure occurs. By transforming drug efficacy assessment from slow and reactive to continuous and predictive, electrochemical biosensors create a foundation for more adaptive, personalized, and successful cancer therapy.

### 7.1. Early Detection of Therapeutic Resistance and Drug Metabolites to Assess Drug Efficacy and Toxicity

The monitoring of therapeutic efficacy, for early detection of resistance, toxicity, or relapse, is essential for clinical oncology. Chemoresistance, driven by genetic and epigenetic alterations, metabolic reprogramming, and survival mechanisms such as enhanced drug efflux [172,173] and activation of alternative signaling pathways, can be a major challenge in cancer treatment. In this context, cutting-edge approaches such as phenotypic screening, analyzing the molecular profile of a patient’s tumor, and pooled CRISPR-based strategies are increasingly employed to uncover previously unidentified targets, mechanisms of resistance, and side effects [174,175]. Current advances in biosensing technologies can revolutionize real-time monitoring of treatment responses and resistance, since biosensors, unlike traditional assays, can swiftly convert biomolecular interactions into electrical signals with high sensitivity and portability, offering immediate insight into the evolving state of disease. As a result, emerging resistance or early signs of toxicity can be identified sooner, moving oncology toward a proactive, personalized approach in which interventions are guided by timely molecular indicators [176,177]. Nevertheless, nowadays, most biosensing platforms are applied towards biomarker detection for diagnosis and prognosis of CRC [90], but there are only a few biosensors proposed for continuous monitoring of chemoresistance or cancer recurrences.

To address limitations of conventional assays, such as low sensitivity, high cost, and long turnaround times, a novel electrochemical DNA biosensor was developed for specific detection of the KRAS G12D mutation. The detection of KRAS mutations is critical for guiding colorectal cancer (CRC) therapy, as these alterations drive resistance to anti-EGFR (epidermal growth factor receptor) treatments. The platform uses an rGO/ZIF-8 nanocomposite synthesized via an in situ growth method, forming a porous, high–surface-area structure that supports efficient probe immobilization and rapid charge transfer. Comprehensive structural and electrochemical characterization confirmed these properties. The biosensor achieved a low detection limit of 35 fM, a broad linear range (100 fM–20 nM), and high selectivity, effectively distinguishing mutant sequences from wild-type and mismatched targets [178]. A nucleic-acid sensor [179] was developed to detect miR-21, a microRNA consistently implicated in colorectal cancer across oncogenesis, proliferation, invasion, and treatment resistance. The platform incorporates fluorescein isothiocyanate (FITC) labeled and biotinylated probes to enable the detection of urinary miR-21, recognized as relevant biomarkers in colorectal cancer, to study disease oncogenesis, proliferation, invasion, and treatment resistance. These probes were immobilized on a modified screen-printed carbon electrode (SPCE), translating target–probe interactions into measurable electrochemical signals using chronoamperometry and cyclic voltammetry. Interleukin-6 (IL-6) is elevated in colorectal cancer (CRC) tissues and serves as a predictive biomarker for stages I–III CRC. High IL-6 levels are associated with advanced disease, poor survival, and tumor-promoting effects mediated through IL-6 trans-signaling, which drives tumor growth, angiogenesis, metastasis, and therapy resistance. To enable sensitive detection of IL-6, researchers developed an LSCF-based interdigitated microelectrode (IDME) sensor using aptamer-conjugated gold nanoparticles. This design enhances aptamer immobilization and allows IL-6 detection down to 1 pg/mL. The sensor also demonstrated high selectivity, successfully measuring IL-6 in spiked serum samples without interference [180].

A MOX [101] nanosensor (SCENT B2) has demonstrated the capability to detect colorectal cancer relapses from patients’ blood samples over a three-year follow-up period, with indications of gender-related variations. Its effectiveness in distinguishing blood from tumor-affected individuals versus healthy subjects—even within a complex follow-up protocol—underscores its potential to strengthen cancer screening strategies and enhance the reliability of patient monitoring. The same approach was applied to detect colorectal cancer biomarkers by gas sensors for supporting clinical follow-up and relapse monitoring [151].

### 7.2. Detection of Circulating Tumor DNA (ctDNA), Exosomes, or Protein Biomarkers as Indicators of Treatment Response

Liquid-biopsy biomarkers such as circulating tumor DNA (ctDNA), exosomes and serum proteins provide dynamic information on tumor burden and therapeutic efficacy in colorectal cancer (CRC) [181,182]. Although clinical monitoring currently depends on laboratory-based assays, recent advances in microfluidic, electrochemical and nanomaterial-enhanced systems have enabled the development of biosensors capable of detecting these biomarkers with the sensitivity and frequency required for real-time therapy monitoring [183]. A number of these devices have been explicitly proposed for postoperative surveillance, recurrence detection or monitoring of treatment progress in CRC.

A notable example is an immunomagnetic bead–integrated microfluidic biosensor designed for rapid isolation of cell-free nucleic acids (including ctDNA) from plasma using dimethyl dithiobispropionimidate (DTBP)–modified magnetic beads [184]. The system enriches cfDNA within approximately fifteen minutes and is explicitly described as suitable for patients at high risk of CRC, postoperative recurrence monitoring and efficacy assessment. Following on-chip isolation, the enriched ctDNA can be analyzed for clinically relevant CRC mutations (e.g., KRAS, BRAF) or methylation patterns, enabling repeated sampling throughout therapy [185]. In parallel, a lateral-flow DNA biosensor has been developed for multiplex detection of KRAS mutations at codons 12 and 13 from synthetic targets, CRC cell lines, tissue specimens and liquid biopsy material [186]. Although initially validated for diagnostic purposes, the platform’s rapid operation and compatibility with circulating DNA support its potential use in serial monitoring of mutant ctDNA dynamics during systemic treatment, where decline or re-emergence of KRAS mutations may reflect therapeutic response or resistance. Broader families of electrochemical ctDNA biosensors, employing nanostructured electrodes and hybridisation probes to detect CRC-associated mutations at femtomolar concentrations, further demonstrate the feasibility of liquid-biopsy biosensing for therapy monitoring [187]. Several studies emphasize that such platforms offer a pathway toward real-time, minimally invasive monitoring of treatment response and minimal residual disease. Despite this promise, most ctDNA biosensors remain at the proof-of-concept stage, and longitudinal CRC studies correlating biosensor readouts with therapeutic outcomes are still limited [187,188].

Exosomes are rich sources of tumor-derived proteins, lipids and nucleic acids, and their cargo changes dynamically with tumor evolution and treatment exposure. An advanced example relevant to CRC is an integrated field-flow fractionation (FFF)–assisted microfluidic biosensor, which separates small extracellular vesicles and quantifies exosomal biomarkers for colon-cancer liquid biopsy [163,189]. Although primarily evaluated for diagnostic use, this device processes small blood volumes and supports repeated measurements of exosomal signatures, making it technically suitable for longitudinal assessment of exosomal proteins or miRNAs associated with chemoresistance [190]. Additional microfluidic and electrochemical exosome biosensors employing aptamers or antibodies against CD63, CD9, CD81, EpCAM, and other tumor markers have demonstrated sensitive detection of tumor-derived exosomes, with several reviews highlighting their value for disease monitoring and evaluation of treatment efficacy [191,192]. Exosomal miRNAs implicated in CRC chemoresistance—including miR-21, miR-17-5p and related oncogenic signatures—represent promising targets for these platforms. While longitudinal therapeutic monitoring in CRC patients has not yet been performed at scale, the underlying technology clearly supports serial analysis suitable for treatment-response assessment.

Protein markers such as carcinoembryonic antigen (CEA) remain widely used for monitoring CRC, and the development of biosensors has significantly improved analytical sensitivity. A representative platform is a microfluidic surface-enhanced Raman scattering (SERS) biosensor using Au@SiO_2_ substrates and Ag nanocubes for the detection of serum biomarkers, including CEA. This device achieves detection limits as low as 0.28 pg/mL and is explicitly proposed for early diagnosis, postoperative monitoring and screening of patients with colorectal cancer [193]. Such sensitivity far exceeds that of standard clinical assays and can, in principle, detect subtle fluctuations in CEA during treatment or early recurrence.

Complementary to this, a smartphone-integrated electrochemical immunosensor based on CuO nanoparticles, carbon nanotubes and graphene oxide has been described for ultra-sensitive, rapid and point-of-care CEA detection. The system achieves sub-pg/mL detection limits and is designed for use in outpatient or low-resource settings, supporting frequent monitoring of CEA kinetics, which are often used as surrogates for tumor burden in CRC management [194].

Multiplex microfluidic immunosensors employing immunomagnetic bead trapping have also been demonstrated for simultaneous measurement of CEA and other serum proteins, with applications described in routine follow-up, postoperative evaluation and repeated monitoring scenarios. Such platforms could be adapted for multi-marker CRC panels (e.g., CEA, CA19-9), potentially increasing the predictive value for therapy response [195,196].

### 7.3. Assessment of Targeted Therapy: Drugs That Target Specific Molecular Changes in Cancer Cells

The HER family of receptors—especially EGFR, HER2, and HER3 [197]—drives key signaling pathways that promote colorectal cancer (CRC) growth and survival, making them important targets for therapies such as cetuximab, panitumumab, trastuzumab, and various HER-targeting tyrosine kinase inhibitors [198,199,200,201]. These treatments have improved outcomes in selected patients, particularly those with RAS wild-type metastatic CRC [202], and ongoing developments such as dual [203]—or pan [204]-HER inhibitors, antibody–drug conjugates, and bispecific antibodies aim to further expand therapeutic options.

Expression of HER family members in colorectal cancer (CRC) is highly variable, with large inconsistencies reported for EGFR, HER2, HER3, and HER4 due to differences in detection methods, antibodies, scoring criteria, tumor heterogeneity, and discordance between primary and metastatic sites. EGFR expression, for example, ranges from 8 to 100% across studies [205,206] and has shown little predictive value for response to anti-EGFR therapies, partly because commonly used assays do not distinguish wild-type from mutated EGFR. HER2 expression is also inconsistent (1.8–70%) and often discordant between primary and metastatic tumors [207,208], though recent work links membranous HER2 to poorer progression-free survival on anti-EGFR treatment [205]. HER3 is frequently expressed and associated with aggressive behavior, anti-EGFR resistance, and activation of compensatory signaling [209], whereas HER4’s role is less clear, showing context-dependent tumor-promoting or tumor-suppressive effects [210]. Furthermore, resistance to targeted therapy remains a major clinical challenge.

Genetic alterations, including KRAS, NRAS, BRAF, and PIK3CA mutations, PTEN loss, and HER2 or MET amplification often lead to primary resistance to anti-EGFR agents [211]. A miniaturized biosensor approach using a lab-on-chip (LoC) device with integrated ion-sensitive field-effect transistors (ISFETs) to run loop-mediated isothermal amplification (LAMP) assays targeting the key CRC biomarker mutation BRAF p.V600E [157] has been developed. The LAMP reactions, both on a regular qPCR machine and on the LoC device, were allele-specific and achieved a limit of detection of ~500 copies/µL and a time-to-positive under 15 min. Parallel control and non-specific reactions exhibited distinct signal trends, and a signal-processing method was developed to rapidly distinguish positive vs. negative outputs. The LoC system proved robust for mutation detection and holds promise for adaptation to multiplexed assays. In addition, another study addressed the challenge of detecting single-nucleotide polymorphisms (SNPs) during cancer therapy, specifically the PIK3CA E545K mutation [212], where the mutant and wild-type DNA differ by only one base. The authors developed a highly selective and sensitive method using locked nucleic acid (LNA)-modified primers combined with hairpin-shaped primers in real-time PCR. They successfully detected the PIK3CA E545K mutation in cell-free DNA with a selectivity of 0.1% and a limit of detection of 10 aM (0.12 fg). The method was validated in clinical samples from a colorectal cancer patient and compared to digital droplet PCR with favorable results. Nonetheless, a novel small-molecule fluorescent probe, Crizotinib-PEG4-MPA has been developed for the specific targeting of the receptor tyrosine kinase c-Met (overexpressed in CRC) for molecular fluorescence imaging [213]. In vitro binding confirmed its specificity with a dissociation constant (Kd) of ~3.86 µM. In vivo in tumor-bearing mice, the tumor/normal signal ratio reached ~4.23 at 6 h. In a colitis-associated colon adenoma model, the probe achieved a significantly higher fluorescence intensity in adenoma versus normal colon (*p* < 0.001). Immunofluorescence of biopsy samples confirmed its targeting of c-Met and its ability to distinguish adenoma from normal tissue.

In addition, non-genetic mechanisms—such as overexpression of EGFR or HER3 ligands that sustain downstream signaling—contribute to acquired resistance [214]. Tumour cells may also compensate by activating HER2 and HER3, enabling bypass signaling despite EGFR inhibition [205].

## 8. Discussion

Colorectal cancer received limited global attention in prior decades but is now recognized as one of the most lethal and prevalent malignancies worldwide. Today, it ranks as the fourth most lethal cancer globally and the third most diagnosed [73]. Although the effectiveness of systematic screening varies greatly by country in Europe and by patient age, several efficient healthcare programs offer early diagnosis and better survival rates than those with advanced cancer. Systematic screening has also reduced mortality rates [148,215]. The majority of patients are around fifty years old, and colorectal cancer is rarely identified before the age of forty. The disease is caused by an adenoma [216]. The majority of young patients have many symptoms, including rectal bleeding, followed by abdominal pain and changes in bowel habits, despite the fact that screening is crucial to the diagnosis [217].

Extensive evidence demonstrates that biological sex significantly influences colorectal cancer (CRC) development and progression. Men are diagnosed more often and earlier than women, while postmenopausal women tend to develop right-sided, MSI-high tumors associated with MLH1 hypermethylation. These sex-specific differences arise from hormonal, molecular, and epigenetic mechanisms that shape tumor biology and immune responses. Recognizing and integrating these distinctions into screening, prevention, and treatment strategies—such as microbiota modulation, hormonal therapy, and targeted immunotherapies—can enhance personalized care. Ultimately, incorporating sex-specific biomarkers into CRC research and clinical practice offers a pathway toward more precise and effective patient management.

The pathogenesis of CRC involves multiple genetic and epigenetic alterations, including mutations in tumor suppressor genes (APC, TP53, SMAD4), oncogenes (KRAS, BRAF), and mismatch repair (MMR) genes [218]. Microsatellite instability (MSI), chromosomal instability (CIN), and CpG island methylator phenotype (CIMP) are key molecular pathways driving tumor progression. Advances in genomic research have enabled molecular classification of CRC, improving personalized treatment strategies. Advances in screening, molecular profiling, and treatment strategies have significantly improved patient outcomes.

Nowadays, research is focused on improving early detection, identifying new therapeutic targets, and advancing precision medicine approaches. Artificial intelligence (AI)-assisted colonoscopy, liquid biopsy for early diagnosis, novel immunotherapeutic agents are promising areas of development and advancements in biosensor technology for cancer detection. Efforts to reduce CRC disparities through public awareness and access to screening remain crucial [219,220].

In this perspective, biosensors have developed into very promising instruments that provide rapid detection of a wide variety of CRC biomarkers at low cost and with high sensitivity [221]. This includes genetic mutations (e.g., KRAS, BRAF), epigenetic changes (e.g., methylated SEPT9, SHOX2) [125], proteins (e.g., carcinoembryonic antigen [CEA]), microRNAs (e.g., miR-21, miR-92a) [222], and volatile organic compounds (VOCs) linked to tumor metabolism. Particularly, electrochemical biosensors have demonstrated extraordinary sensitivity and specificity when combined with nanomaterials like polypyrrole, gold nanoparticles, and carbon nanotubes, which improve electron transfer and the immobilization of biomolecules [223,224].

Recent developments in VOC-based biosensing offer a new non-invasive method for CRC diagnostics by analyzing exhaled breath or fecal emissions [81]. These methods take advantage of the changed metabolic environment of cancer cells and their relationship with gut microbiota, providing a diagnostic solution that is both patient-friendly and scalable [225]. Nanosensors have also enhanced detection limits and made it possible to create portable, multiplexed platforms that are appropriate for point-of-care (POC) applications [226]. In addition, biosensors are considered cost-effective tools by minimizing dependence on pricey imaging like MRI or CT scans, extensive lab setups, and skilled staff. They support point-of-care testing with tiny reagent amounts and promote early detection to cut future treatment expenses. Plus, their disposable, mass-producible nature drives down costs per test dramatically [227].

Several biosensors have undergone clinical validation across diverse medical applications, highlighting their translational potential in healthcare. A noninvasive tear-glucose sensor demonstrated clinical feasibility as an alternative to finger-prick testing for diabetes [228]. A fiber-optic SPR biosensor tested plasma from ten individuals to identify breast-cancer–specific extracellular vesicles [229] while a piezoelectric quartz biosensor detected alpha-fetoprotein in patient serum samples. Electrochemical biosensors have also been clinically validated for the detection of methylglyoxal in subjects with type-2 diabetes [230]. In oncology, a saliva-based electrochemical/printed circuit board (PCB) biosensor detected breast cancer biomarkers HER2 and CA15-3 in 29 human saliva samples with ultra-high sensitivity, effectively distinguishing disease states [231]. An electrochemical biosensor for miRNA-21 was tested on 40 human clinical samples (lung cancer patients and healthy controls), successfully differentiating cancerous from non-cancerous specimens [232]. Similarly, an electrochemical miRNA-31 biosensor was evaluated in human serum samples for oral cancer detection, demonstrating high sensitivity and specificity [233]. For colorectal cancer, clinical validation has been reported in various cases, such as a nanostructured gas sensor using 398 fecal samples from Fecal Immunochemical Test-positive individuals, achieving 84.1% and 82.4% specificity compared with colonoscopy [234]. An electrochemical biosensor based on a covalent organic framework probe was developed for detecting CRC-derived exosomes. It demonstrated excellent analytical performance with a low detection limit of 160 particles/μL and was successfully applied to clinical serum samples [235]. Additionally, an electronic nose (eNose) breath sensor was tested in 105 CRC patients and 186 non-cancer controls, distinguishing CRC from controls with an area under the curve (AUC) of approximately 0.734 [236]. Collectively, these studies underscore the growing clinical support for biosensors in noninvasive diagnostics and cancer detection [237].

Although the recent advances in biosensor technologies have demonstrated their ability to detect complex cancer biomarkers with high analytical sensitivity, several challenges must be addressed before these platforms can be broadly adopted in clinical practice [218]. Most evaluations to date have shown exceptional analytical performance only under controlled laboratory conditions often in small, single-center cohorts [238]), and have not fully accounted for matrix effects in clinical samples [239] or variables such as patient demographics, sex, and age [240,241]. These limitations hinder cross-platform comparability and restrict the evidence required to establish clinical utility [242,243]. Additional challenges include differences in sample preparation, the absence of standardization, sensor stability, and selectivity in complex biological fluids [219,220]. The lack of standardized protocols, calibration procedures, and variability in fabrication methods further complicates clinical translation. Regulatory approval demands rigorous demonstration of safety, efficacy, and clinical utility through well-designed clinical trials, which involve complex validation processes [244]. Interdisciplinary collaboration among biologists, chemists, physicists, engineers, and data specialists is essential but presents logistical challenges. Integration with digital health platforms, such as cloud systems and mobile applications, adds another layer of complexity and remains a major research focus [245]. Most biosensors have been validated in cross-sectional or early translational studies, but large-scale longitudinal trials directly linking bios—particularly in colorectal cancer—are still lacking. The necessity of sex-stratified biosensor validation highlights a key direction for future research, especially as the field moves toward precision real-time monitoring of cancer therapy. To overcome these barriers, harmonized validation frameworks, standardized sample handling and calibration procedures, and large, multicenter prospective trials benchmarking biosensors against established diagnostic workflows are increasingly needed [246,247,248]. By aligning research designs with regulatory requirements for analytical and clinical validity, and by demonstrating clear clinical utility across diverse patient cohorts, next-generation biosensors hold significant potential to transform early cancer detection and personalized oncology diagnostics [249,250,251].

Colorectal cancer continues to pose a major global health challenge, as increasing incidence and death rates highlight the urgent requirement for effective and widely available early diagnostic methods [6,252]. The timely diagnosis of colorectal cancer is fundamental to its management, as early-stage detection is strongly associated with better treatment outcomes and higher survival rates [253]. Nonetheless, the current screening methods, although they work well, often have drawbacks related to sensitivity, invasiveness, cost, and patient compliance [254]. This has led to an urgent need for the creation of innovative diagnostic tools that are minimally invasive and highly sensitive [255]. New technologies such as molecular biomarker assays, biosensors, and non-invasive sampling methods show great promise for advancing CRC diagnostics [256]. The goal of these advancements is to improve detection accuracy and reduce procedural burden, but also to enhance patient compliance and increase access to screening, especially in low-resource environments [9]. To fully harness the potential of these new diagnostic modalities and to effectively address the global burden of colorectal cancer, ongoing interdisciplinary research, technological innovation, and clinical validation are crucial [257].

## Figures and Tables

**Table 1:** Non-modifiable and modifiable risk factors for colorectal cancer.

Non Modifiable Risk Factors	Modifiable Risk Factors
Ethnicity	Sedentary lifestyle
Sex	Obesity
Age	Diet
Hereditary mutations	Smoking
Inflammatory bowel diseases	Alcohol consumption
Cystic fibrosis	Diabetes and insulin resistance
Acromegaly	Gut microbiota
Cholecystectomy	
Treatment with androgen derivatives	

**Table 2:** AJCC Colorectal Cancer Staging Table (8th Edition) [35].

Stage	Tumor (T)	Lymph Nodes (N)	Metastasis (M)	Description
0	Tis	N0	M0	Early cancer limited to the innermost lining (carcinoma in situ).
I	T1 or T2	N0	M0	Tumor has penetrated the inner layers of the bowel wall but not the lymph nodes or distant organs.
IIA	T3	N0	M0	Tumor has extended into the outer layers of the colon/rectum without breaking through.
IIB	T4a	N0	M0	Tumor has broken through the outer wall but has not invaded nearby organs.
IIC	T4b	N0	M0	Tumor has grown into or adhered to nearby structures or organs.
IIIA	T1–T2 or T1	N1/N1c or N2a	M0	Tumor has invaded inner layers and spread to a few nearby lymph nodes or surrounding fat.
IIIB	T3–T4a or T2–T3 or T1–T2	N1/N1c or N2a or N2b	M0	Tumor extends through outer layers and has spread to multiple nearby lymph nodes.
IIIC	T4a or T3–T4a or T4b	N2a or N2b or N1/N2	M0	Advanced local tumor spread and significant lymph node involvement, but no distant metastasis.
IVA	Any T	Any N	M1a	Cancer has spread to a single distant organ or lymph node group.
IVB	Any T	Any N	M1b	Cancer has spread to more than one distant organ or lymph node group.
IVC	Any T	Any N	M1c	Cancer has spread to the lining of the abdominal cavity (peritoneum), possibly with other distant spread.

**Table 4:** Major colorectal cancer (CRC) diagnostic methodologies.

Method	Description	Time to Result	Purpose	Notes
**Fecal Occult Blood Test (FOBT)** [75]	A stool-based test detecting heme in hemoglobin via guaiac resin reaction. Requires dietary restrictions to reduce false positives.	1–7 days	Early detection of hidden (occult) blood indicating possible CRC or polyps.	Annual screening; limited sensitivity multiple samples needed.
**Fecal Immunochemical Test (FIT)** [9]	Uses antibodies to detect human hemoglobin; more specific to lower GI bleeding. No dietary restrictions required.	1–3 days	Preferred stool-based test for early CRC detection.	Annual screening; higher specificity than gFOBT; single sample sufficient.
**Colonoscopy** [75]	Direct visualization of colon via a flexible scope. Allows detection and removal of polyps and biopsy. High-definition imaging and chromoendoscopy can enhance detection.	Immediate (preliminary); biopsy results: 3–7 days	Gold standard for both screening and diagnosis of CRC and colonic diseases.	Every 10 years for average risk; requires bowel prep and sedation; risks include perforation and bleeding.
**Flexible Sigmoidoscopy** [9]	Endoscopic exam of rectum and lower colon (up to ~60 cm). No sedation is typically required. Can remove polyps and biopsy tissue.	Immediate (preliminary); biopsy results: 3–7 days	Screening tool especially for distal colon lesions.	Less invasive than colonoscopy; performed every 5 years. Limited to lower colon.

## Data Availability

No new data were created or analyzed in this study. Data sharing is not applicable to this article.

## Abbreviations

The following abbreviations are used in this manuscript:

ADR	Adenoma Detection Rate
AI	Artificial Intelligence
AJCC	American Joint Committee on Cancer
APC	Adenomatous Polyposis Coli
AuNPs	Gold Nanoparticles
Au@SiO_2_	Gold-Coated Silica Nanoparticles
BRAF	v-Raf Murine Sarcoma Viral Oncogene Homolog B1
CA	Carbohydrate Antigen
CA19-9	Carbohydrate Antigen 19-9
CEA	Carcinoembryonic Antigen
CFU	Colony Forming Unit
CLIA	Chemiluminescent Immunoassay
CNT	Carbon Nanotube
CNTs	Carbon Nanotubes
CuO	Copper Oxide
CRC	Colorectal Cancer
CRISPR	Clustered Regularly Interspaced Short Palindromic Repeats
CRP	C-Reactive Protein
CT	Computed Tomography
ctDNA	Circulating Tumor DNA
CTCs	Circulating Tumor Cells
DNA	Deoxyribonucleic Acid
DTBP	Dimethyl Dithiobispropionimidate
EGFR	Epidermal Growth Factor Receptor
EIS	Electrolyte–Insulator–Semiconductor
EpCAM	Epithelial Cell Adhesion Molecule
ERβ	Estrogen Receptor Beta
EV	Extracellular Vesicle
FAP	Familial Adenomatous Polyposis
FET	Field-Effect Transistor
FFF	Field-Flow Fractionation
FIT	Fecal Immunochemical Test
FITC	Fluorescein Isothiocyanate
FOBT	Fecal Occult Blood Test
GC	Gas Chromatography
GFET	Graphene Field-Effect Transistor
GO	Graphene Oxide
HER	Human Epidermal Growth Factor Receptor Family
HER2	Human Epidermal Growth Factor Receptor 2
HER3	Human Epidermal Growth Factor Receptor 3
HER4	Human Epidermal Growth Factor Receptor 4
HEMT	High Electron Mobility Transistor
HNPCC	Hereditary Nonpolyposis Colorectal Cancer
HRP	Horseradish Peroxidase
IHC	Immunohistochemistry
IDME	Interdigitated Microelectrode
IL-6	Interleukin 6
ISFET	Ion-Sensitive Field-Effect Transistor
KRAS	Kirsten Rat Sarcoma Viral Oncogene Homolog
Kd	Dissociation Constant
LAMP	Loop-Mediated Isothermal Amplification
LED	Light Emitting Diode
LNA	Locked Nucleic Acid
LoC	Lab-on-a-Chip
LSPR	Localized Surface Plasmon Resonance
LSCF	Lanthanum Strontium Cobalt Ferrite
miRNA	MicroRNA
MMR	Mismatch Repair
MOX	Metal Oxide (Sensor)
MRI	Magnetic Resonance Imaging
MSI	Microsatellite Instability
MSI-H	Microsatellite Instability High
MT	Microtubule
MXene	Transition Metal Carbide/Nitride (2D Material)
NIR	Near-Infrared
NK	Natural Killer (Cell)
NMOF	Nanoscale Metal–Organic Framework
PDMS	Polydimethylsiloxane
PEG	Polyethylene Glycol
PET	Positron Emission Tomography
PIK3CA	Phosphatidylinositol-4,5-Bisphosphate 3-Kinase Catalytic Subunit Alpha
QCM	Quartz Crystal Microbalance
qPCR	Quantitative Polymerase Chain Reaction
RAS	Rat Sarcoma (Oncogene Family)
RNA	Ribonucleic Acid
rGO	Reduced Graphene Oxide
SCENT B2	Metal-Oxide Nanosensor Platform (Product Name)
SERS	Surface-Enhanced Raman Scattering
SNP	Single-Nucleotide Polymorphism
SPCE	Screen-Printed Carbon Electrode
SPR	Surface Plasmon Resonance
TDM	Therapeutic Drug Monitoring
TGF	Transforming Growth Factor
TILs	Tumor-Infiltrating Lymphocytes
TNM	Tumor Node Metastasis
VEGF	Vascular Endothelial Growth Factor
VOC	Volatile Organic Compound
VTSB	Vision-Based Tactile Sensing Balloon
ZIF-8	Zeolitic Imidazolate Framework-8
c-Met	Mesenchymal–Epithelial Transition Factor (Receptor Tyrosine Kinase)
